# Transplantation Without Overimmunosuppression (TWO) study protocol: a phase 2b randomised controlled single-centre trial of regulatory T cell therapy to facilitate immunosuppression reduction in living donor kidney transplant recipients

**DOI:** 10.1136/bmjopen-2022-061864

**Published:** 2022-04-15

**Authors:** Matthew Oliver Brook, Joanna Hester, William Petchey, Ines Rombach, Susan Dutton, Matthew James Bottomley, Joanna Black, Seetha Abdul-Wahab, Andrew Bushell, Giovanna Lombardi, Kathryn Wood, Peter Friend, Paul Harden, Fadi Issa

**Affiliations:** 1 Nuffield Department of Surgical Sciences, University of Oxford, Oxford, UK; 2 Oxford Transplant Centre, Oxford University Hospitals NHS Foundation Trust, Oxford, UK; 3 Nuffield Department of Orthopaedics, Rheumatology and Musculoskeletal Sciences, University of Oxford, Oxford, UK; 4 NIHR Biomedical Research Centre GMP unit, Guy’s Hospital, London, UK; 5 Peter Gorer Department of Immunobiology, King’s College London Faculty of Life Sciences and Medicine, London, UK

**Keywords:** Transplant surgery, TRANSPLANT MEDICINE, Clinical trials, IMMUNOLOGY, Nephrology

## Abstract

**Introduction:**

Regulatory T cell (Treg) therapy has been demonstrated to facilitate long-term allograft survival in preclinical models of transplantation and may permit reduction of immunosuppression and its associated complications in the clinical setting. Phase 1 clinical trials have shown Treg therapy to be safe and feasible in clinical practice. Here we describe a protocol for the TWO study, a phase 2b randomised control trial of Treg therapy in living donor kidney transplant recipients that will confirm safety and explore efficacy of this novel treatment strategy.

**Methods and analysis:**

60 patients will be randomised on a 1:1 basis to Treg therapy (TR001) or standard clinical care (control). Patients in the TR001 arm will receive an infusion of autologous polyclonal ex vivo expanded Tregs 5 days after transplantation instead of standard monoclonal antibody induction. Maintenance immunosuppression will be reduced over the course of the post-transplant period to low-dose tacrolimus monotherapy. Control participants will receive a standard basiliximab-based immunosuppression regimen with long-term tacrolimus and mycophenolate mofetil immunosuppression. The primary endpoint is biopsy proven acute rejection over 18 months; secondary endpoints include immunosuppression burden, chronic graft dysfunction and drug-related complications.

**Ethics and dissemination:**

Ethical approval has been provided by the National Health Service Health Research Authority South Central—Oxford A Research Ethics Committee (reference 18/SC/0054). The study also received authorisation from the UK Medicines and Healthcare products Regulatory Agency and is being run in accordance with the principles of Good Clinical Practice, in collaboration with the registered trials unit Oxford Clinical Trials Research Unit. Results from the TWO study will be published in peer-reviewed scientific/medical journals and presented at scientific/clinical symposia and congresses.

**Trial registration number:**

ISRCTN: 11038572; Pre-results.

**Strengths and limitations of this study**

- Randomisation will provide a contemporary control group to compare outcomes following regulatory T cell therapy and immunosuppression minimisation.
- Absence of an induction agent, day 5 regulatory T cell infusion and protocol defined immunosuppression reduction to low dose tacrolimus monotherapy in the TR001 arm represents a significant reduction in pharmaceutical immunosuppression burden compared with standard care.
- Comprehensive clinical and immune monitoring planned over an 18-month follow-up will permit assessment of clinical safety and efficacy as well as exploration of markers of immune activation and tolerance.
- This study may be limited through being an open-label single-centre trial.
- As a phase 2b trial with small participant numbers in each arm this study is not powered to provide definitive proof of efficacy.

## Introduction

Kidney transplantation is the gold standard treatment for patients with end-stage kidney disease and is associated with excellent short-term outcomes with graft survival of greater than 95% for living donor transplant recipients at 1 year.^1^ However, there remains significant scope for improvement in long-term outcomes with progressive reduction in graft survival over time.^1^ Furthermore, outcomes are limited by the complications of immunosuppression such as life-threatening infection, increased cardiovascular disease risk and malignancy.^2–5^ Novel treatments such as regulatory T cell (Treg) therapy may improve long-term patient and graft outcomes both by reducing immune mediated graft dysfunction and facilitating reduction of immunosuppression to minimise the associated side effects.^6–8^

Tregs are typically defined by expression of the cell surface markers being CD4^+^CD25^+^ and their constitutive expression of the master transcription factor FOXP3. Extensive preclinical models have demonstrated their potency at suppressing rejection responses resulting in long-term allograft survival in the absence of pharmaceutical immunosuppression.^9–11^ The first steps in translation of Treg therapy into the clinical setting of organ transplantation were taken by Todo *et al* who infused a Treg enriched cell product (less than 15% Treg) into liver transplant recipients.^12^ Seven of 10 patients were able to completely withdraw immunosuppression although 3 patients experienced rejection episodes. The low purity of Tregs in the infused cell product and incidence of spontaneous tolerance in liver transplant recipients makes interpretation of these results uncertain. In kidney transplantation, we have recently demonstrated successful infusion of autologous polyclonal Tregs into 12 patients recruited as part of the ONE study consortium.^13 14^ This phase 1 trial used dose escalation from 3×10^6^ to 10×10^6^ Tregs/kg bodyweight infused at day 5 post transplantation. Participants did not receive any monoclonal antibody induction therapy and were initially maintained on prednisolone, mycophenolate mofetil and tacrolimus. Immunosuppression was weaned over the course of the first year and 4 of 12 patients were ultimately successfully reduced to tacrolimus monotherapy. Four-year follow-up demonstrated no episodes of rejection compared with a 21.1% rejection rate in a retrospective control cohort receiving standard care. Furthermore, there was a suggestion of reduced incidence of opportunistic cytomegalovirus (CMV) and BKV infections.^13^ Our ONE study colleagues in Berlin infused 11 patients with autologous polyclonal Tregs in a dose escalation manner at day 7 post transplant.^15^ Eight patients were weaned successfully to tacrolimus monotherapy. Three of 11 patients experienced biopsy proven acute rejection, a rate similar to that seen in patients undergoing standard care.^15^ These studies have demonstrated initial safety and feasibility of Treg therapy and provide justification for continuation into phase 2 trials.^14^

The TWO study will build on our work performed as part of the ONE study consortium^14^ to provide further evidence of safety and to explore efficacy of Treg therapy to facilitate immunosuppression reduction in living donor kidney transplant recipients.

The TWO study was originally conceived as a phase 2b randomised (1:1) control trial of Treg therapy versus standard care in 68 living donor kidney transplant recipients. Patients in both arms received standard alemtuzumab induction at the point of transplant to facilitate lymphodepletion with a view to optimising the environment into which Treg were later infused in favour of tolerance induction.^16^ Immunosuppression in the Treg arm was minimised to tacrolimus monotherapy in advance of cell infusion at 6 months post transplant and compared with ongoing standard maintenance immunosuppression with tacrolimus and mycophenolate mofetil. Target tacrolimus levels were reduced in the cell therapy arm to 4–6 ng/mL from week 40 post transplant. The primary outcome was incidence of biopsy proven acute rejection between 6 and 18 months post transplant.

Nine patients were recruited to this protocol and seven transplanted prior to the emergence of the COVID-19 pandemic. Due to concerns related to an increased risk of severe COVID-19 in the setting of alemtuzumab lymphodepletion, the trial protocol was modified to one using basiliximab-based induction immunosuppression. Basiliximab is a widely used induction immunosuppressive agent that binds to and blocks CD25, the alpha chain of the interleukin (IL)-2 receptor, resulting in T cell suppression. Seven patients treated under the original protocol with alemtuzumab induction will be reported as a cohort demonstrating our experience of Treg administration in this context. The current protocol comparing Treg therapy to basiliximab-based standard immunosuppression will recruit 60 participants, form the basis of the TWO study and is reported in detail here.

## Methods and analysis

### Patient and public involvement

Patients were involved in the design and conduct of the TWO study. During development the proposed study was presented and discussed with a patient focus group to ensure that it addressed a relevant need to the transplant patient community. Methodology was discussed to ensure acceptability and address any concerns. A transplant recipient has joined the independent trial steering committee (TSC) bringing an invaluable patient perspective to discussions. Once the trial has been published, participants will be informed of the outcomes directly and results will be distributed to relevant patient groups.

### Study design

**Figure 1:**
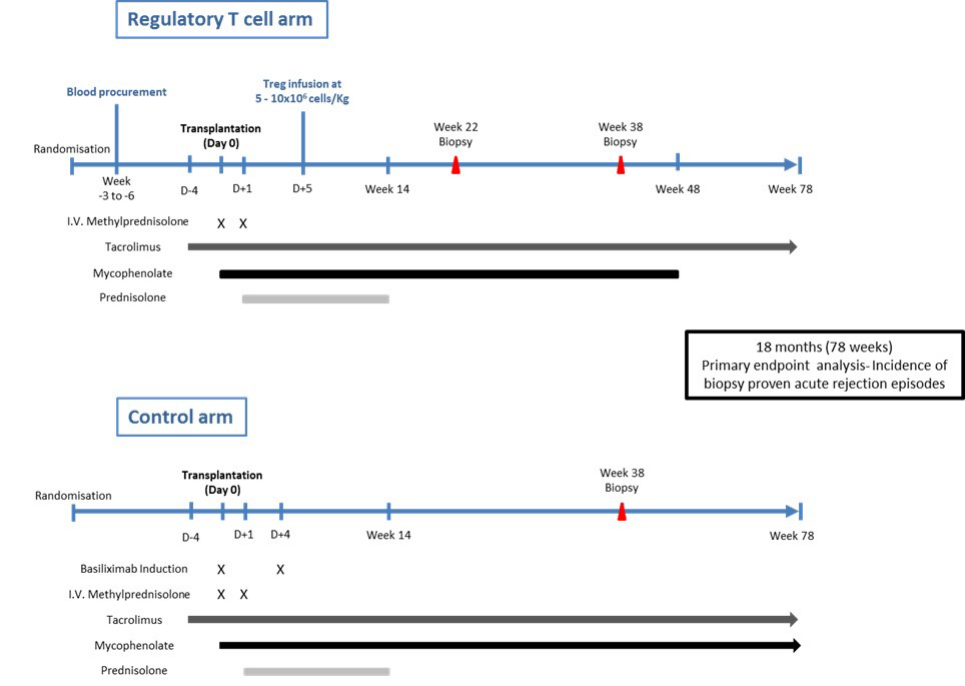
Diagrammatic representation of the immunosuppressive regimen used in The TWO study.

In this parallel group, phase IIb trial, 60 eligible living donor kidney transplant recipients will be recruited from that undergoing kidney transplantation at a single academic hospital (Oxford Transplant Centre, Churchill Hospital, Oxford, UK) and randomised on a 1:1 basis to receive a standard basiliximab-based immunosuppressive regimen (control arm) or Treg infusion associated with immunosuppression reduction (TR001 arm) (figure 1).

Participants will be approached and enrolled by the clinical principal investigator (PI) or deputy following approval of listing for living donor kidney transplantation by the clinical multidisciplinary team meeting. Randomisation is computer generated and performed by minimisation, with stratification for ethnicity and Human Leukocyte Antigen - DR (HLA-DR) mismatch. Treatment allocation will be open-label as pre-transplant venesection of blood for Treg manufacture in those allocated to the TR001 arm is required and it is not ethically appropriate to perform venesection in control patients prior to major surgery. Accordingly, outcome assessors and statisticians are not blinded.

With a relatively small patient sample size, the emergence of significant numbers of patient discontinuation in the trial may obscure the true outcome of this research. Discontinued participants may be replaced by the recruitment of additional patients. The decision to replace individual patients will ultimately be made by the clinical PI on the basis that some unanticipated factor may influence the clinical outcome in terms of the primary endpoint.

### Inclusion and exclusion criteria

Inclusion and exclusion criteria for both kidney transplant recipient and donor are listed in box 1. Specific to transplantation, exclusion criteria originally included a calculated reaction frequency (cRF) of >40% and a history of previous transplant. These were subsequently amended to permit recipients with a cRF of <60% and to allow patients with a previous transplant to participate. ABO blood group incompatible transplants, the presence of a pre-transplant donor specific antibodies (DSA), or a history of desensitisation continue to meet exclusion criteria to ensure those transplants with the highest immunological risk are not included in this phase IIb study.

Box 1 **Inclusion and exclusion criteria for TWO study transplant recipients and donors**

**Kidney recipient inclusion criteria**

A prospective kidney transplant recipient is eligible for enrolment into the study if all of the following inclusion criteria apply:

- Chronic renal insufficiency necessitating kidney transplantation and approved to receive a kidney allograft from a living donor.
- Willing and able to give informed consent for participation in the trial.
- Aged 18 years or above.
- In the investigator’s opinion, is able and willing to comply with all trial requirements.
- Able to commence the immunosuppressive regimen at the protocol-specified time point.
- Female participants of child bearing potential and male participants whose partner is of child bearing potential must be willing to ensure that they or their partner use highly effective contraception during the first 18 months post transplant (see section on contraception).
- Willing to allow his or her general practitioner and consultant, if appropriate, to be notified of participation in the trial.

**Kidney recipient exclusion criteria**

The participant may not enter the trial if ANY of the following apply:

- Patient has previously received any tissue or organ transplant.*
- Known contraindication to the protocol-specified treatments or medications.
- ABO blood group incompatible with donor.
- Calculated reaction frequency (cRF) of >60%† within 6 months prior to transplant.
- Previous treatment with any desensitisation procedure (with or without IVIg).
- Concomitant malignancy or history of malignancy within 5 years prior to planned study entry (excluding successfully treated non-metastatic basal or squamous cell carcinomas of the skin).
- Serologically positive for anti-HIV-1/2 Ab, HbsAg, anti-HBcAb, anti-HCV Ab, anti-HTLV-1/2 Ab or syphilis (treponema palladium).
- Significant liver disease, defined as persistently elevated Alanine Aminotransferease levels >3 × upper limit of normal range.
- Any other significant disease or disorder which, in the opinion of the investigator, may either put the participants at risk because of participation in the trial, or may influence the result of the trial, or the participant’s ability to participate in the trial.
- Participation in another clinical trial during the study or within 28 days prior to planned study entry.
- Female participant who is pregnant, lactating or planning pregnancy during the course of the trial.
- Psychological, familial, sociological or geographical factors potentially hampering compliance with the study protocol and follow-up visit schedule.
- Any form of substance abuse, psychiatric disorder or other condition.

**Kidney donor inclusion criteria**

A prospective donor is eligible if all of the following inclusion criteria apply:

- Eligible for live kidney donation.
- Aged at least 18 years.
- ABO blood group compatible with the organ recipient.
- Willing to provide personal, medical and biological data for the trial analysis.
- Willing and able to provide a blood sample for the immune monitoring assays.
- Willing and able to give informed consent for participation in the trial.

**Kidney donor exclusion criteria**

If a prospective donor fulfils any of the following criteria, they are ineligible for the trial:

- Exposure to any investigational agents at the time of kidney donation, or within 28 days prior to kidney donation.
- Any form of substance abuse, psychiatric disorder or other condition that, in the opinion of the investigator, may invalidate communication with the investigator designated personnel.
- Is a paired exchange donor.
- Is an altruistic donor.

### Control arm

**Figure 2:**
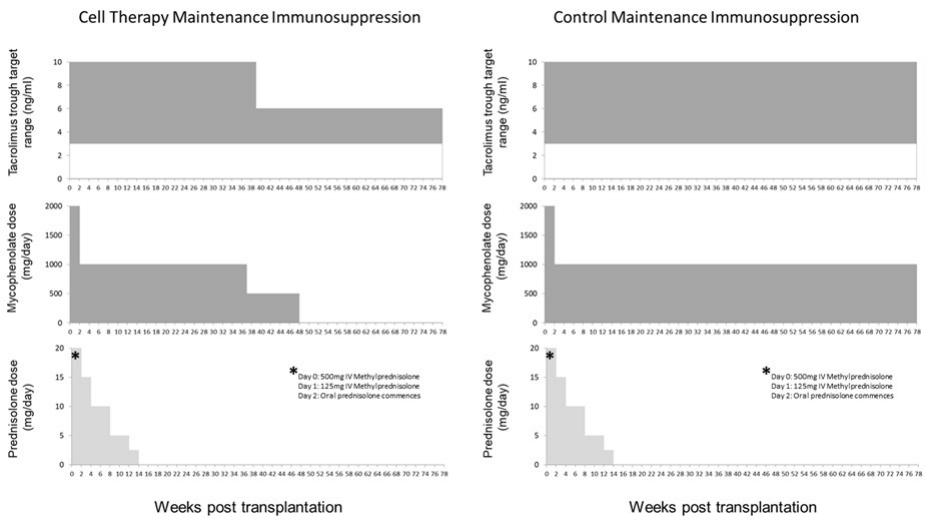
Overview of maintenance immunosuppression dosing with minimisation in the TR001 (cell therapy) arm.

Participants in the control arm undergo planned living donor kidney transplantation with a standard basiliximab (anti-CD25) based immunosuppression protocol (figure 1). Briefly, patients will be preloaded with tacrolimus starting 4 days prior to transplantation and continued long-term aiming for trough levels of 3–10 ng/mL. On the day of transplant patients commence mycophenolate mofetil at an initial maintenance dose of 1000 mg two times a day. Intravenous methylprednisolone 500 mg and intravenous basiliximab 20 mg are administered at induction. On day 1 post transplant 125 mg of intravenous methylprednisolone is administered before ongoing oral prednisolone commences at 20 mg one time a day on day 2. A further 20 mg of intravenous basiliximab is given on day 4 post transplant. Maintenance immunosuppression on discharge thus consists of tacrolimus aiming for trough levels of 3–10 ng/mL, mycophenolate mofetil 1000 mg two times a day and prednisolone 20 mg one time a day. Mycophenolate mofetil is reduced to 500 mg two times a day from 14 days post transplant and continued long-term. Prednisolone is weaned to stop over 14 weeks resulting in dual maintenance therapy with mycophenolate mofetil and tacrolimus. Immunosuppression regimens and dose reductions in both arms are summarised in figure 2.

### TR001 arm

Patients recruited to the cell therapy arm attend for venesection of 370 mL of whole blood a minimum of 3 weeks prior to planned transplantation to permit manufacture of the autologous Treg product (TR001). Following transport to the good manufacturing practice unit at Guy’s and St Thomas’ Hospital, London, whole blood undergoes negative selection of CD8^+^ cells and positive selection of CD25^+^ cells resulting in enrichment of CD4^+^CD25^+^FOXP3^+^ Treg (approximately 75% of total cells entering the expansion phase). Polyclonal expansion of cells is achieved through up to three rounds of stimulation with anti-CD3 and anti-CD28 bead stimulation in the presence of IL-2. Importantly, rapamycin is added to the culture conditions and has been shown to promote Treg stability and preferential expansion over contaminant populations. Full details of the expansion protocol have been described elsewhere.^17^ Following expansion, the final cell product is cryopreserved at a dose of 5–10×10^6^ cells/kg body weight of the intended recipient in preparation for future infusion.

Living donor kidney transplantation occurs in line with standard clinical practice but with minimisation of immunosuppression from the outset in the TR001 arm. Initial maintenance immunosuppression with tacrolimus (Envarsus, Chiesi is the preferred long-acting sustained release formulation in both arms to avoid Treg toxicity that may occur at peak concentrations), mycophenolate mofetil and prednisolone is provided in an identical manner to those participants in the control arm. Importantly, where basiliximab is administered to control patients, those in the TR001 arm will receive no monoclonal induction agent at the time of transplantation. On day 5 post-transplant patients in the TR001 arm receive an infusion of 5–10×10^6^ cells/kg of thawed autologous polyclonal Tregs administered in 100 mL of 5% human albumin solution.

Planned reduction of maintenance immunosuppression in the TR001 arm will be dependent on stable biochemical transplant function. In the TR001 arm, protocol biopsies are performed for monitoring purposes at 22 weeks and 38 weeks post transplant. Target trough tacrolimus levels are reduced from 3 to 10 ng/mL to 3–6 ng/mL at week 38 once biopsy results have been received. The maintenance dose of mycophenolate mofetil will be reduced to 250 mg two times a day from week 37 post transplant and stopped at 48 weeks post transplant such that patients will subsequently continue on low-dose tacrolimus monotherapy as long-term maintenance (figure 2).

### Primary outcome

The primary outcome is incidence of biopsy-confirmed acute rejection (BCAR) in the 18 months post transplantation. A diagnosis of BCAR can be made based on protocol driven or clinically indicated ‘for cause’ biopsies. ‘For cause’ biopsies may be performed during follow-up at the discretion of the responsible clinician taking into account the full clinical picture and are typically triggered by an unexplained rise in serum creatinine as per standard National Health Service (NHS) practice. Whenever rejection is suspected, a for-cause graft biopsy will always be offered and performed with the patient’s permission. The results of for-cause biopsies will be available to the trial investigators and the outcome will be documented in the electronic database.

All biopsies performed will be reviewed and reported by the study pathologist using the internationally accepted Banff criteria. Whenever a biopsy is reported as suspicious for rejection or borderline changes, responsibility for a diagnosis of rejection lies with the treating physician.

### Secondary outcomes

**Figure 3:**
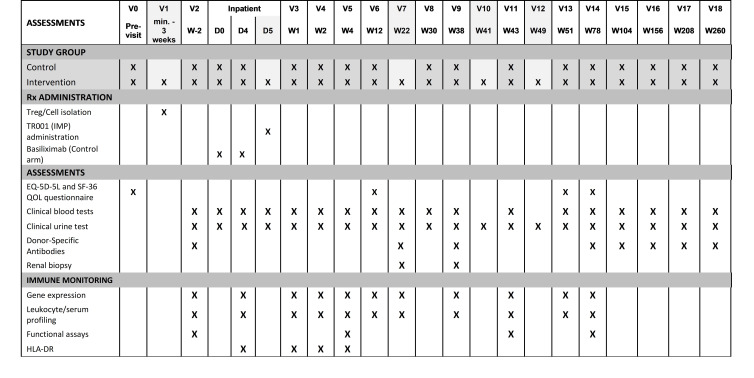
Key time points alongside clinical and immune monitoring plans. EQ-5D-5L, EuroQol 5 Dimensions 5 Levels; HLA-DR, Human Leukocyte Antigen - DR Isotype; IMP, investigational medicinal product; QOL, Quality of Life; SF-36, 36 Item Short Form Survey; Treg, regulatory T cell.

A number of secondary outcomes are defined in order to assess the safety, feasibility and potential additive benefits of both cellular therapy and associated immunosuppression minimisation on the clinical course of recipients post transplantation (figure 3). These secondary outcomes will be continuously monitored throughout the 18-month follow-up period post transplantation unless otherwise stated and can be further defined as follows:

#### Indicators of influence of Treg administration on graft outcome

Impact on acute rejection: Time to first acute rejection episode; severity of acute rejection episode based on response to treatment and histological scoring; total immunosuppressive burden at the final trial visit; and incidence of graft loss through rejection.

Success in reduction of immunosuppression: Proportion of patients on tacrolimus monotherapy at the end of the study.

Prevention of chronic graft dysfunction: Assessment of renal impairment, chronic allograft dysfunction and/or interstitial fibrosis and tubular atrophy assessed by clinical (impairment of estimated glomerular filtration rate (eGFR)) and histopathological (Banff staging) measures.

Avoidance of drug-related complications by immunosuppressant reduction: Incidence of drug-related adverse events.

### Patient survival

#### Markers of oversuppression of the immune system

Incidence of serious and/or opportunistic infections (especially CMV, Epstein Barr virus (EBV) and polyoma (BK) virus) and incidence of neoplasia.

#### Signs of chronic toxicity associated with infusion of cell products

Incidence of autoimmune disorders, anaemia, cytopenias or biochemical disturbances unrelated to the function of the transplanted kidney.

#### Patient quality of life

Patient quality of life will be measured in both arms of the study at pre-transplant baseline, 12 weeks, 51 weeks and 78 weeks post transplant using the 36 Item Short Form Survey (SF-36) and EuroQol 5 Dimensions 5 Levels (EQ-5D-5L) questionnaire.

### Immune monitoring

A critical component of the TWO study is comprehensive assessment of the impact of Treg infusion on the recipient’s immune repertoire and its capacity to respond to donor, third-party and non-allogeneic stimuli. Importantly, these assays will include analysis of whole blood and transplant biopsy samples taken from patients in both arms of the study. Assays remain experimental and will not be used to influence clinical decision-making in the TWO study. However, accumulating evidence suggests the potential for these tools in tailoring individualised immunosuppression regimens and we aim to identify those that might prove suitable for this purpose going forwards while providing important mechanistic information on a basic science level in the current study. Figure 3 provides an overview of immune monitoring assays being performed.

Absolute quantification of HLA-DR expression by peripheral blood monocytes is a useful and reproducible surrogate marker of innate immune responses. HLA-DR quantification will be performed by flow cytometry and interpreted using the following pre-determined ranges: Normal healthy controls >15 000 molecules per cell; immunodepression 15 000–8000 molecules per cell; immunoparesis <8000 molecules per cell.

Assays will be performed to investigate whether cell therapy shifts kidney transplant recipients towards a more tolerance-prone phenotype or away from a rejection-prone phenotype. Gene expression of a defined set of tolerance-associated genes in whole blood will be profiled by quantitative PCR. Leucocyte subset profiling will be performed by flow cytometry to quantify immune cell subpopulations in patient peripheral blood. Donor-reactive T cell frequencies will be measured following co-culture of recipient T cells with stored donor derived antigen presenting cells using a CD154/137 assay. This assay will be performed before and after transplantation to enable an estimation of the pre-transplant frequency of donor-reactive T cells, and detection of post-transplant sensitisation against donor antigen. Treg frequencies in patient blood will be measured by epigenetic analysis of the Treg-specific demethylated region of the FOXP3 gene. Finally, cytokine and metabolic profiling will be performed assessing inflammatory and regulatory cytokines as well as low-molecular-weight metabolites to provide a picture of the dynamic changes that may take place in the immune response after cellular therapy and immunosuppression modification.

Histopathological samples will be taken at 5 months (protocol biopsy) in kidney transplant recipients randomised to the TR001 arm. This biopsy will confirm the ongoing safety of Treg therapy and ensure no evidence of subclinical rejection. A 9-month protocol biopsy will be performed in all participants including the control arm to allow a histological comparison of the impact of Treg therapy.

### Sample size calculation

A standard anti-CD25 monoclonal antibody-based immunosuppression protocol as used in this study would be expected to result in a biopsy proven acute rejection rate of approximately 12%–20% over 18 months post transplant. Ekberg *et al* demonstrated that daclizumab induction with triple maintenance therapy of low-dose tacrolimus, myophenolate mofetil and corticosteroids resulted in acute rejection diagnoses in 12.3% of transplant recipients in the first year post transplant, a significant improvement on comparable alternative regimens at the time.^18^ Recently, the 3C study reported a 16% acute rejection rate in the first 6 months of a basiliximab-based immunosuppression regimen and a further 3% over the following 18 months up to 2 years post transplant.^19 20^ There is little data on anticipated rejection rates in patients treated with Treg therapy. We reported in our phase 1 trial a rejection rate of 21.1% in a control cohort receiving basiliximab-based immunosuppression compared with no rejection episodes in patients receiving Treg therapy over 60 weeks post transplant.^13^ In contrast, Roemchild *et al*, demonstrated a rejection rate of 27% in patients treated with polyclonal Treg therapy and 22% in an identical control cohort.^15^ However, numbers were small in both studies and although both used autologous polyclonal Treg the manufacturing processes and quality control assessment of the final product differed.

The TWO study is a phase 2b study aimed at proving the feasibility, ongoing safety and exploring the efficacy of Treg therapy to facilitate a reduction in standard immunosuppression. We aim to provide the data required for future phase 3 sample size calculations. Recruitment of 30 participants in each arm will allow us to estimate rejection rates in both arms with an anticipated 80% Wilson CI width between 10% and 23%, depending on the observed rate.

### Data analysis plan

This early phase study will report data using 20% statistical significance and 80% CIs.

Two analysis sets will be defined:

- Intention-to-treat population: all patients who signed informed consent and were transplanted will be analysed in the groups to which they were randomised.
- Per-protocol population: all patients who signed informed consent, were transplanted and were treated according to protocol specifications.

Descriptive statistics will be used to describe the demographics between the treatment groups. Withdrawn patients will also be described fully. Comparative analysis will be undertaken to provide an indication as to whether a definitive phase 3 randomised trial would be appropriate.

For continuous variables, the difference in the means and the corresponding 80% CI will be reported for each treatment group and overall. For continuous variables, t-tests unadjusted or multivariable linear models adjusted for important factors will be applied.

For categorical variables, the number (and percentage) of patients in each category will be reported for each treatment group and overall. For categorical variables, χ^2^ tests will be used for comparing treatment groups or multivariable logistic models adjusted for important factors.

The primary outcome is biopsy proven acute rejection episode and the time to first biopsy proven acute rejection will be analysed using survival analysis techniques. Kaplan-Meier survival curves will be presented graphically. Cox proportional hazards models will be used both unadjusted and adjusted for important factors. The log-rank test will be used to identify significance. Acute rejection rates at 18 months will be reported for both groups and as a difference in proportions, alongside the HRs and 80% CI will be reported. Patients who have been withdrawn or lost to follow-up will be censored at their last known rejection-free time. Analysis adjusting for competing risks of allograft failure or death will be considered.

No interim analyses are planned, but a data safety and monitoring committee (DSMC) will review descriptive summaries of accumulating data and make recommendations on trial termination or modification to the TSC based on these data. The independent members of the DSMC panel are chosen from those leading in the field of clinical transplantation and/or with experience of previous cell therapy trials in the ONE study consortium. They will conduct a review of data at least annually at the discretion of the committee and will be informed of any serious adverse reactions (SARs) or suspected unexpected serious adverse reactions (SUSARs) as they occur by email notification. The DSMC charter is available from the TWO study team.

## Ethics, governance and dissemination

This manuscript is based on TWO study protocol V.7.0, 11 August 2020. The TWO study has received ethical approval from NHS Health Research Authority South Central—Oxford A Research Ethics Committee (reference 18/SC/0054). In addition, the study has received authorisation from the UK Medicines and Healthcare products Regulatory Agency.

All information, data and results obtained from the TWO study are confidential. Agreement from the sponsor and TSC will be required prior to the public disclosure of any study-related data.

The results from the TWO study will be published in peer-reviewed scientific/medical journals and presented at scientific/clinical symposia and congresses.

## Supplementary Material

Reviewer comments

Author's manuscript
