# Supplementary material for: Transplantation Without Overimmunosuppression (TWO) study protocol: a phase 2b randomised controlled single-centre trial of regulatory T cell therapy to facilitate immunosuppression reduction in living donor kidney transplant recipients
Source: BMJ Open. 2022 Apr 15;12(4):e061864. doi: 10.1136/bmjopen-2022-061864 (PMC9014059; doi:10.1136/bmjopen-2022-061864)
Supplement: Author's manuscript [file bmjopen-2022-061864.draft_revisions.pdf]

BMJ Open

BMJ Open is committed to open peer review. As part of this commitment we make the peer review history of every article we publish publicly available.

When an article is published we post the peer reviewers' comments and the authors' responses online. We also post the versions of the paper that were used during peer review. These are the versions that the peer review comments apply to.

The versions of the paper that follow are the versions that were submitted during the peer review process. They are not the versions of record or the final published versions. They should not be cited or distributed as the published version of this manuscript.

BMJ Open is an open access journal and the full, final, typeset and author-corrected version of record of the manuscript is available on our site with no access controls, subscription charges or pay-per-view fees (http://bmjopen.bmj.com).

If you have any questions on BMJ Open's open peer review process please email info.bmjopen@bmj.com

BMJ Open

Transplantation Without Overimmunosuppression (TWO) Study: A phase 2b randomised controlled single-centre trial of regulatory T cell therapy to facilitate immunosuppression reduction in living donor kidney transplant recipients

Journal:	*BMJ Open*
Manuscript ID	bmjopen-2022-061864
Article Type:	Protocol
Date Submitted by the Author:	09-Feb-2022
Complete List of Authors:	Brook, Matthew; University of Oxford Nuffield Department of Surgical Sciences; Oxford Transplant Centre,<br>Hester, Joanna; University of Oxford Nuffield Department of Surgical Sciences<br>Petchey, William; Oxford Transplant Centre,<br>Rombach, Ines; University of Oxford Nuffield Department of Orthopaedics Rheumatology and Musculoskeletal Sciences<br>Dutton, Susan; University of Oxford Nuffield Department of Orthopaedics Rheumatology and Musculoskeletal Sciences<br>Bottomley, Matthew; University of Oxford Nuffield Department of Surgical Sciences; Oxford Transplant Centre,<br>Black, Joanna; University of Oxford Nuffield Department of Orthopaedics Rheumatology and Musculoskeletal Sciences<br>Bushell, Andrew; University of Oxford Nuffield Department of Surgical Sciences<br>Lombardi, Giovanna; King's College London Faculty of Life Sciences and Medicine, Peter Gorer Department of Immunobiology<br>Wood, Kathryn; University of Oxford Nuffield Department of Surgical Sciences<br>Friend, Peter; University of Oxford Nuffield Department of Surgical Sciences; Oxford Transplant Centre,<br>Harden, Paul; Oxford Transplant Centre,<br>Issa, Fadi; University of Oxford Nuffield Department of Surgical Sciences
Keywords:	Transplant surgery < SURGERY, TRANSPLANT MEDICINE, Clinical trials < THERAPEUTICS, IMMUNOLOGY, Nephrology < INTERNAL MEDICINE

SCHOLARONE™
Manuscripts

Transplantation Without Overimmunosuppression (TWO) Study: A phase 2b randomised controlled single-centre trial of regulatory T cell therapy to facilitate immunosuppression reduction in living donor kidney transplant recipients

Matthew Brook[1,2], Joanna Hester[1], William Petchey[2], Ines Rombach[3], Susan Dutton[3], Matthew Bottomley[1,2], Joanna Black[3], Andrew Bushell[1], Giovanna Lombardi[4], Kathryn Wood[1], Peter Friend[2], Paul Harden[2]*, Fadi Issa[1]*.

1. Transplantation Research & Immunology Group, Nuffield Department of Surgical Sciences, University of Oxford, John Radcliffe Hospital, Oxford, U.K.
2. Oxford Transplant Centre, Oxford University Hospitals NHS Foundation Trust, Churchill Hospital, Oxford, U.K.
3. Oxford Clinical Trials Research Unit, Nuffield Orthopaedic Centre, Windmill Road, Headington, Oxford, U.K.
4. Peter Gorer Department of Immunobiology, School of Immunology and Microbial Science, Kings College London, London, UK

*Equal contribution to work

Corresponding author:

Dr Matthew Brook, Oxford Kidney Unit, Churchill Hospital, Oxford, OX3 7LE

+447411521634

matthew.brook@ouh.nhs.uk

Keywords:

Regulatory T cell

Renal Transplantation

Immune Tolerance

Living Donors

Immunosuppression minimisation

Word Count: 3752

For peer review only - http://bmjopen.bmj.com/site/about/guidelines.xhtml

ABSTRACT

Introduction

Regulatory T cell (Treg) therapy has been demonstrated to facilitate long-term allograft survival in pre-clinical models of transplantation and may permit reduction of immunosuppression and its associated complications in the clinical setting. Phase 1 clinical trials have shown Treg therapy to be safe and feasible in clinical practice. Here we describe a protocol for the TWO Study, a phase 2b randomised control trial of Treg therapy in living donor kidney transplant recipients that will confirm safety and explore efficacy of this novel treatment strategy.

Methods and Analysis

60 patients will be randomised on a 1:1 basis to Treg therapy (TR001) or standard clinical care (Control). Patients in the TR001 arm will receive an infusion of autologous polyclonal ex-vivo expanded Tregs 5 days after transplantation instead of standard monoclonal antibody induction. Maintenance immunosuppression will be reduced over the course of the post-transplant period to low-dose tacrolimus monotherapy. Control participants will receive a standard basiliximab-based immunosuppression regimen with long-term tacrolimus and mycophenolate mofetil immunosuppression. The primary endpoint is biopsy proven acute rejection over 18 months; secondary endpoints include immunosuppression burden, chronic graft disfunction, and drug-related complications.

Ethics and Dissemination

Ethical approval has been provided by the NHS Health Research Authority South Central - Oxford A Research Ethics Committee (reference 18/SC/0054). The study also received authorisation from the UK MHRA and is being run in accordance with the principles of good clinical practice (GCP), in collaboration with the registered trials unit OCTRU. Results from the TWO Study will be published in peer-reviewed scientific/medical journals and presented at scientific/clinical symposia and congresses.

The TWO Study is registered on the ISRCTN registry (11038572).

For peer review only

For peer review only - http://bmjopen.bmj.com/site/about/guidelines.xhtml

STRENGTHS AND LIMITATIONS OF THIS STUDY

- First phase 2b randomised control trial of regulatory T cell therapy in living-donor kidney transplantation
- Absence of induction agent, Day 5 Treg infusion and immunosuppression reduction to low dose tacrolimus monotherapy in TR001 arm
- Comprehensive clinical and immune monitoring planned over 18 month follow up
- Unblinded single centre trial
- Phase 3 trial will be required for definitive efficacy analysis

INTRODUCTION

Kidney transplantation is the gold standard treatment for patients with end-stage kidney disease and is associated with excellent short-term outcomes with graft survival of greater than 95% for living donor transplant recipients at 1 year(1). However, there remains significant scope for improvement in long-term outcomes with progressive reduction in graft survival over time(1). Furthermore, outcomes are limited by the complications of immunosuppression such as life-threatening infection, increased cardiovascular disease risk, and malignancy(2–5). Novel treatments such as regulatory T cell (Treg) therapy may improve long-term patient and graft outcomes both by reducing immune mediated graft dysfunction and facilitating reduction of immunosuppression to minimise the associated side-effects(6–8)

Tregs are typically defined by expression of the cell surface markers being $CD4^{+}CD25^{+}$ and their constitutive expression of the master transcription factor FOXP3. Extensive pre-clinical models have demonstrated their potency at supressing rejection responses resulting in long-term allograft survival in the absence of pharmaceutical immunosuppression(9–11). The first steps in translation of Treg therapy into the clinical setting of organ transplantation were taken by Todo et al. who infused a Treg enriched cell product (less than 15% Treg) into liver transplant recipients(12). 7 of 10 patients were able to completely withdraw immunosuppression although 3 patients experienced rejection episodes. The low purity of Tregs in the infused cell product and incidence of spontaneous tolerance in liver transplant recipients makes interpretation of these results uncertain. In kidney transplantation, we have recently demonstrated successful infusion of autologous polyclonal Tregs into 12 patients recruited as part of the ONE Study consortium(13,14). This phase 1 trial used dose escalation from $3x10^{6}$ to $10x10^{6}$ Tregs/kg bodyweight infused at day 5 post-transplantation. Participants did not receive any monoclonal antibody induction therapy and were initially maintained on prednisolone, mycophenolate mofetil and tacrolimus. Immunosuppression was weaned over the course of the first year and 4 of 12 patients were ultimately successfully reduced to tacrolimus monotherapy. 4 year follow up demonstrated no episodes of rejection compared to a 21.1% rejection rate in a retrospective control cohort receiving standard care. Furthermore, there was a suggestion of reduced incidence of opportunistic CMV and BKV infections (13). Our ONE Study colleagues in Berlin infused 11 patients with autologous polyclonal Tregs in a dose escalation manner at day 7 post-transplant(15). 8 patients were weaned successfully to tacrolimus monotherapy. 3 of 11 patients experienced biopsy proven acute rejection, a rate similar to that seen in patients undergoing standard care(15). These studies have demonstrated initial safety and feasibility of Treg therapy and provide justification for continuation into phase 2 trials(14).

The TWO study will build on our work performed as part of the ONE study consortium(14) to provide further evidence of safety and to explore efficacy of Treg therapy to facilitate immunosuppression reduction in living donor kidney transplant recipients.

For peer review only - http://bmjopen.bmj.com/site/about/guidelines.xhtml

The TWO study was originally conceived as a phase 2b randomised (1:1) control trial of Treg therapy versus standard care in 68 living donor kidney transplant recipients (ISRCTN: 11038572). Patients in both arms received standard alemtuzumab induction at the point of transplant to facilitate lymphodepletion with a view to optimising the environment into which Treg were later infused in favour of tolerance induction(16). Immunosuppression in the Treg arm was minimised to tacrolimus monotherapy in advance of cell infusion at 6 months post-transplant and compared to ongoing standard maintenance immunosuppression with tacrolimus and mycophenolate mofetil. Target tacrolimus levels were reduced in the cell therapy arm to 4-6 ng/mL from week 40 post-transplant. The primary outcome was incidence of biopsy proven acute rejection between 6 and 18 months post-transplant.

Nine patients were recruited to this protocol and seven transplanted prior to the emergence of the COVID-19 pandemic. Due to concerns related to an increased risk of severe COVID-19 in the setting of alemtuzumab lymphodepletion, the trial protocol was modified to one utilising basiliximab-based induction immunosuppression. Basiliximab is a widely used induction immunosuppressive agent that binds to and blocks CD25, the alpha chain of the IL-2 receptor, resulting in T cell suppression. Seven patients treated under the original protocol with alemtuzumab induction will be reported as a cohort demonstrating our experience of Treg administration in this context. The current protocol comparing Treg therapy to basiliximab based standard immunosuppression will recruit 60 participants, form the basis of the TWO study and is reported in detail here.

METHODS AND ANALYSIS

Patient and public involvement

Patients were involved in the design and conduct of the TWO Study. During development the proposed study was presented and discussed with a patient focus group to ensure that it addressed a relevant need to the transplant patient community. Methodology was discussed to ensure acceptability and address any concerns. A transplant recipient has joined the independent trial steering committee bringing an invaluable patient perspective to discussions. Once the trial has been published, participants will be informed of the outcomes directly and results will be distributed to relevant patient groups.

Study design

In this parallel group, phase IIb trial, 60 eligible living donor kidney transplant recipients will be recruited from that undergoing kidney transplantation at a single academic hospital (Oxford Transplant Centre, Churchill Hospital, Oxford, U.K.) and randomised on a 1:1 basis to receive a standard basiliximab based immunosuppressive regimen (Control Arm) or Treg infusion associated with immunosuppression reduction (TR001 Arm) (figure 1).

Participants will be approached and enrolled by the clinical PI or deputy following approval of listing for living donor kidney transplantation by the clinical multi-disciplinary team meeting. Randomisation is computer generated and performed by minimisation, with stratification for ethnicity and HLA-DR mismatch. Treatment allocation will be open-label as pre-transplant venesection of blood for Treg manufacture in those allocated to the TR001 arm is required and it is not ethically appropriate to perform venesection in control patients prior to major surgery. Accordingly, outcome assessors and statisticians are not blinded.

With a relatively small patient sample size, the emergence of significant numbers of patient discontinuation in the trial may obscure the true outcome of this research. Discontinued participants may be replaced by the recruitment of additional patients. The decision to replace

For peer review only - http://bmjopen.bmj.com/site/about/guidelines.xhtml

individual patients will ultimately be made by the Clinical PI on the basis that some unanticipated factor may influence the clinical outcome in terms of the primary endpoint.

Inclusion and Exclusion criteria

Inclusion and exclusion criteria for both kidney transplant recipient and donor are listed in Table 1. Specific to transplantation, exclusion criteria originally included a cRF of >40% and a history of previous transplant. These were subsequently amended to permit recipients with a cRF of <60% and to allow patients with a previous transplant to participate. ABO blood group incompatible transplants, the presence of a pre-transplant DSA, or a history of desensitisation continue to meet exclusion criteria to ensure those transplants with the highest immunological risk are not included in this phase IIb study.

Kidney Recipient Inclusion Criteria

A prospective kidney transplant recipient is eligible for enrolment into the study if all of the following inclusion criteria apply:

- Chronic renal insufficiency necessitating kidney transplantation and approved to receive a kidney allograft from a living donor
- Willing and able to give informed consent for participation in the trial
- Aged 18 years or above
- In the Investigator's opinion, is able and willing to comply with all trial requirements
- Able to commence the immunosuppressive regimen at the protocol-specified time point
- Female participants of child bearing potential and male participants whose partner is of child bearing potential must be willing to ensure that they or their partner use highly effective contraception during the first 18 months post-transplant (see section on Contraception)
- Willing to allow his or her General Practitioner and consultant, if appropriate, to be notified of participation in the trial.

Kidney Recipient Exclusion Criteria

The participant may not enter the trial if ANY of the following apply:

- ~~Patient has previously received any tissue or organ transplant~~*
- Known contraindication to the protocol-specified treatments or medications
- ABO blood group incompatible with donor
- Calculated reaction frequency (CRF) of >60%** within 6 months prior to transplant
- Previous treatment with any desensitisation procedure (with or without IVIg)
- Concomitant malignancy or history of malignancy within 5 years prior to planned study entry (excluding successfully treated non-metastatic basal or squamous cell carcinomas of the skin)
- Serologically positive for anti-HIV-1/2 Ab, HbsAg, anti-HBcAb, antiHCV Ab, anti-HTLV-1/2 Ab or syphilis (treponema palladium)
- Significant liver disease, defined as persistently elevated ALT levels >3 x upper limit of normal range (ULN)
- Any other significant disease or disorder which, in the opinion of the Investigator, may either put the participants at risk because of participation in the trial, or may influence the result of the trial, or the participant's ability to participate in the trial

For peer review only

For peer review only - http://bmjopen.bmj.com/site/about/guidelines.xhtml

- Participation in another clinical trial during the study or within 28 days prior to planned study entry
- Female participant who is pregnant, lactating or planning pregnancy during the course of the trial
- Psychological, familial, sociological, or geographical factors potentially hampering compliance with the study protocol and follow-up visit schedule
- Any form of substance abuse, psychiatric disorder, or other condition

*= Removed from exclusion criteria by substantial amendment
**=Changed from >40% by substantial amendment based on new information comparing cRF to historical PRA.

Kidney Donor Inclusion Criteria

A prospective donor is eligible if all of the following inclusion criteria apply:

- Eligible for live kidney donation
- Aged at least 18 years
- ABO blood group compatible with the organ recipient
- Willing to provide personal, medical and biological data for the trial analysis
- Willing and able to provide a blood sample for the immune monitoring assays
- Willing and able to give informed consent for participation in the trial

Kidney Donor Exclusion Criteria

If a prospective donor fulfils any of the following criteria, they are ineligible for the trial:

- Exposure to any investigational agents at the time of kidney donation, or within 28 days prior to kidney donation
- Any form of substance abuse, psychiatric disorder, or other condition that, in the opinion of the Investigator, may invalidate communication with the Investigator designated personnel
- Is a paired exchange donor
- Is an altruistic donor

Table 1: Inclusion and exclusion criteria for TWO study transplant recipients and donors

Control Arm

Participants in the control arm undergo planned living donor kidney transplantation with a standard basiliximab (anti-CD25) based immunosuppression protocol (figure 1). Briefly, patients will be pre-loaded with tacrolimus starting four days prior to transplantation and continued long-term aiming for trough levels of 3-10ng/ml. On the day of transplant patients commence mycophenolate mofetil at an initial maintenance dose of 1000mg twice a day. 500mg of intravenous methylprednisolone and 20mg intravenous basiliximab are administered at induction. On day 1 post-transplant 125mg intravenous methylprednisolone is administered before ongoing oral prednisolone commences at 20mg once a day on day 2. A further 20mg of intravenous basiliximab is given on day 4 post-transplant. Maintenance immunosuppression

For peer review only - http://bmjopen.bmj.com/site/about/guidelines.xhtml

on discharge thus consists of tacrolimus aiming for trough levels of 3-10ng/ml, mycophenolate mofetil 1000mg twice a day and prednisolone 20mg once a day. Mycophenolate mofetil is reduced to 500mg twice a day from 14 days post-transplant and continued long-term. Prednisolone is weaned to stop over 14 weeks resulting in dual maintenance therapy with mycophenolate mofetil and tacrolimus. Immunosuppression regimens and dose reductions in both arms are summarised in figure 2.

TR001 Arm

Patients recruited to the cell therapy arm attend for venesection of 370mls of whole blood a minimum of 3 weeks prior to planned transplantation to permit manufacture of the autologous Treg product (TR001). Following transport to the good manufacturing practice (GMP) unit at Guy's and St Thomas' Hospital, London, whole blood undergoes negative selection of $CD8^+$ cells and positive selection of $CD25^+$ cells resulting in enrichment of $CD4^+CD25^+FOXP3^+$ Treg (approx. 75% of total cells entering the expansion phase). Polyclonal expansion of cells is achieved through up to 3 rounds of stimulation with anti-CD3 and anti-CD28 bead stimulation in the presence of IL-2. Importantly, rapamycin is added to the culture conditions and has been shown to promote Treg stability and preferential expansion over contaminant populations. Full details of the expansion protocol have been described elsewhere(17). Following expansion, the final cell product is cryopreserved at a dose of 5-10x10^6 cells/kg body weight of the intended recipient in preparation for future infusion.

Living donor kidney transplantation occurs in line with standard clinical practice but with minimisation of immunosuppression from the outset in the TR001 arm. Initial maintenance immunosuppression with tacrolimus (Envarsus, Chiesi is the preferred long-acting sustained release formulation in both arms to avoid Treg toxicity that may occur at peak concentrations), mycophenolate mofetil and prednisolone is provided in an identical manner to those participants in the control arm. Importantly, where basiliximab is administered to control patients, those in the TR001 arm will receive no monoclonal induction agent at the time of transplantation. On day 5 post-transplant patients in the TR001 arm receive an infusion of 5-10x10^6 cells/kg of thawed autologous polyclonal Tregs administered in 100mls of 5% human albumin solution (HAS).

Planned reduction of maintenance immunosuppression in the TR001 arm will be dependent on stable biochemical transplant function. In the TR001 arm, protocol biopsies are performed for monitoring purposes at 22 weeks and 38 weeks post-transplant. Target trough tacrolimus levels are reduced from 3-10ng/ml to 3-6ng/ml at week 38 once biopsy results have been received. The maintenance dose of mycophenolate mofetil will be reduced to 250mg twice a day from week 37 post-transplant and stopped at 48 weeks post-transplant such that patients will subsequently continue on low-dose tacrolimus monotherapy as long-term maintenance (figure 2).

Primary Outcome

The primary outcome is incidence of biopsy-confirmed acute rejection (BCAR) in the 18-months post-transplantation. A diagnosis of BCAR can be made based on protocol driven or clinically indicated 'for cause' biopsies. 'For cause' biopsies may be performed during follow-up at the discretion of the responsible clinician taking into account the full clinical picture and are typically triggered by an unexplained rise in serum creatinine as per standard NHS practice. Whenever rejection is suspected, a for-cause graft biopsy will always be offered and performed

For peer review only - http://bmjopen.bmj.com/site/about/guidelines.xhtml

with the patient's permission. The results of for-cause biopsies will be available to the trial investigators and the outcome will be documented in the electronic database.

All biopsies performed will be reviewed and reported by the study pathologist using the internationally accepted Banff criteria. Whenever a biopsy is reported as suspicious for rejection or borderline changes, responsibility for a diagnosis of rejection lies with the treating physician.

Secondary Outcomes

A number of secondary outcomes are defined in order to assess the safety, feasibility and potential additive benefits of both cellular therapy and associated immunosuppression minimisation on the clinical course of recipients post-transplantation (Figure 3). These secondary outcomes will be continuously monitored throughout the 18 month follow-up period post transplantation unless otherwise stated and can be further defined as follows:

<u>Indicators of influence of Treg administration on graft outcome</u>

Impact on acute rejection: Time to first acute rejection episode; Severity of acute rejection episode based on response to treatment and histological scoring; Total immunosuppressive burden at the final trial visit; and Incidence of graft loss through rejection.

Success in reduction of immunosuppression**:** Proportion of patients on tacrolimus monotherapy at the end of the study

Prevention of chronic graft dysfunction: Assessment of renal impairment, chronic allograft dysfunction and/or interstitial fibrosis and tubular atrophy (IF/TA) assessed by clinical (impairment of eGFR) and histopathological (Banff staging) measures

Avoidance of drug-related complications by immunosuppressant reduction: Incidence of drug-related adverse events

Patient survival

<u>Markers of over-suppression of the immune system</u>

Incidence of serious and/or opportunistic infections (especially CMV, EBV and polyoma (BK) virus) and incidence of neoplasia.

<u>Signs of chronic toxicity associated with infusion of cell products</u>

Incidence of auto-immune disorders, anaemia, cytopaenias, or biochemical disturbances unrelated to the function of the transplanted kidney.

<u>Patient quality of life</u>

Patient quality of life will be measured in both arms of the study at pre-transplant baseline, 12 weeks, 51 weeks and 78 weeks post-transplant using SF-36 & EQ-5D-5L questionnaires.

Immune monitoring

A critical component of the TWO study is comprehensive assessment of the impact of Treg infusion on the recipient's immune repertoire and its capacity to respond to donor, third-party and non-allogeneic stimuli. Importantly, these assays will include analysis of whole blood and transplant biopsy samples taken from patients in both arms of the study. Assays remain experimental and will not be used to influence clinical decision making in the TWO study. However, accumulating evidence suggests the potential for these tools in tailoring

For peer review only - http://bmjopen.bmj.com/site/about/guidelines.xhtml

individualised immunosuppression regimens and we aim to identify those that might prove suitable for this purpose going forwards whilst providing important mechanistic information on a basic science level in the current study. Figure 3 provides an overview of immune monitoring assays being performed.

Absolute quantification of HLA-DR expression by peripheral blood monocytes is a useful and reproducible surrogate marker of innate immune responses. HLA-DR quantification will be performed by flow cytometry and interpreted using the following pre-determined ranges: Normal healthy controls >15,000 molecules per cell; immunodepression 15,000 – 8,000 molecules per cell; immunoparesis <8,000 molecules per cell.

Assays will be performed to investigate whether cell therapy shifts kidney transplant recipients towards a more tolerance-prone phenotype or away from a rejection-prone phenotype. Gene expression of a defined set of tolerance-associated genes in whole blood will be profiled by qPCR. Leucocyte subset profiling will be performed by flow cytometry to quantify immune cell subpopulations in patient peripheral blood. Donor-reactive T cell frequencies will be measured following co-culture of recipient T cells with stored donor derived antigen presenting cells using a CD154/137 assay. This assay will be performed before and after transplantation to enable an estimation of the pre-transplant frequency of donor-reactive T cells, and detection of post-transplant sensitisation against donor antigen. Treg frequencies in patient blood will be measured by epigenetic analysis of the Treg-specific demethylated region (TSDR) of the FOXP3 gene. Finally, cytokine and metabolic profiling will be performed assessing inflammatory and regulatory cytokines as well as low-molecular-weight metabolites to provide a picture of the dynamic changes that may take place in the immune response after cellular therapy and immunosuppression modification.

Histopathological samples will be taken at 5 months (protocol biopsy) in kidney transplant recipients randomised to the TR001 arm. This biopsy will confirm the ongoing safety of Treg therapy and ensure no evidence of subclinical rejection. A 9 month protocol biopsy will be performed in all participants including the control arm to allow a histological comparison of the impact of Treg therapy.

Sample size calculation

A standard anti-CD25 monoclonal antibody based immunosuppression protocol as used in this study would be expected to result in a biopsy proven acute rejection rate of approximately 12 to 20% over 18 months post-transplant. Ekberg et al. demonstrated that daclizumab induction with triple maintenance therapy of low-dose tacrolimus, myophenolate mofetil and corticosteroids resulted in acute rejection diagnoses in 12.3% of transplant recipients in the first year post-transplant, a significant improvement on comparable alternative regimens at the time(18). Recently, the 3C study reported a 16% acute rejection rate in the first 6 months of a basiliximab based immunosuppression regimen and a further 3% over the following 18 months up to 2 years post-transplant(19,20). There is little data on anticipated rejection rates in patients treated with Treg therapy. We reported in our phase 1 trial a rejection rate of 21.1% in a control cohort receiving basiliximab based immunosuppression compared with no rejection episodes in patients receiving Treg therapy over 60 weeks post-transplant(13). In contrast, Roemchild et al, demonstrated a rejection rate of 27% in patients treated with polyclonal Treg therapy and 22% in an identical control cohort(15). However, numbers were small in both studies and although both used autologous polyclonal Treg the manufacturing processes and quality control assessment of the final product differed.

For peer review only - http://bmjopen.bmj.com/site/about/guidelines.xhtml

The TWO Study is a phase 2b study aimed at proving the feasibility, ongoing safety and exploring the efficacy of Treg therapy to facilitate a reduction in standard immunosuppression. We aim to provide the data required for future phase 3 sample size calculations. Recruitment of 30 participants in each arm will allow us to estimate rejection rates in both arms with an anticipated 80% Wilson confidence interval width between 10-23%, depending on the observed rate.

Data analysis plan

This early phase study will report data using 20% statistical significance and 80% confidence intervals.

Two analysis sets will be defined:

- Intention to-treat population: all patients who signed informed consent and were transplanted will be analysed in the groups to which they were randomised
- Per-protocol population: all patients who signed informed consent, were transplanted and were treated according to protocol specifications.

Descriptive statistics will be used to describe the demographics between the treatment groups. Withdrawn patients will also be described fully. Comparative analysis will be undertaken to provide an indication as to whether a definitive phase 3 randomised trial would be appropriate.

For continuous variables, the difference in the means and the corresponding 80% confidence interval will be reported for each treatment group and overall. For continuous variables, t-tests unadjusted or multivariable linear models adjusted for important factors will be applied

For categorical variables, the number (and percentage) of patients in each category will be reported for each treatment group and overall. For categorical variables, chi-squared tests will be used for comparing treatment groups or multivariable logistic models adjusted for important factors.

The primary outcome is biopsy proven acute rejection episode and the time to first biopsy proven acute rejection will be analysed using survival analysis techniques. Kaplan-Meier survival curves will be presented graphically. Cox proportional Hazards models will be used both unadjusted and adjusted for important factors. The log-rank test will be used to identify significance. Acute rejection rates at 18 months will be reported for both groups and as a difference in proportions, alongside the hazard ratios and 80% confidence interval will be reported. Patients who have been withdrawn or lost-to follow-up will be censored at their last known rejection-free time. Analysis adjusting for competing risks of allograft failure or death will be considered.

No interim analyses are planned, but a data safety and monitoring committee (DSMC) will review descriptive summaries of accumulating data and make recommendations on trial termination or modification to the trial steering committee (TSC) based on these data. The independent members of the DSMC panel are chosen from those leading in the field of clinical transplantation and/or with experience of previous cell therapy trials in the ONE Study consortium. They will conduct a review of data at least annually at the discretion of the committee and will be informed of any SARs or SUSARs as they occur by e-mail notification. The DSMC charter is available from the TWO Study team.

For peer review only - http://bmjopen.bmj.com/site/about/guidelines.xhtml

ETHICS, GOVERNANCE AND DISSEMINATION

This manuscript is based on TWO Study protocol version 7.0 11Aug2020. The TWO Study has received ethical approval from NHS Health Research Authority South Central - Oxford A Research Ethics Committee (reference 18/SC/0054). In addition, the study has received authorisation from the UK MHRA.

All information, data and results obtained from the TWO Study are confidential. Agreement from the Sponsor and TSC will be required prior to the public disclosure of any study-related data.

The results from the TWO Study will be published in peer-reviewed scientific/medical journals and presented at scientific/clinical symposia and congresses.

The TWO Study is sponsored by the University of Oxford (ctrg@admin.ox.ac.uk).

Authors' contributions:

PJF is chief investigator

PH & FI are Co-Principal investigators

FI is the MRC grant holder

JB, MB, SD, PF, JH, PH, FI, GL, WP, IR & KW contributed to development of the initial protocol

JH has oversight of immune monitoring activities

All authors have contributed to amendments made to the initial study protocol

Funding statement:

This work was supported by Medical Research Council grant number MR/N027930/1.

Competing interests statement:

PH is an advisor to Sangamo Therapeutics

Envarsus long-acting sustained release tacrolimus is provided by Chiesi Ltd on a cost-neutral basis.

MJB has received an educational grant and speakers fees from Astellas UK (Ltd)

REFERENCES

1. Poggio ED, Augustine JJ, Arrigain S, Brennan DC, Schold JD. Long-term kidney transplant graft survival—Making progress when most needed. American Journal of Transplantation [Internet]. 2021 Aug 8;21(8):2824–32. Available from: https://onlinelibrary.wiley.com/doi/10.1111/ajt.16463

2. Rama I, Grinyó JM. Malignancy after renal transplantation: the role of immunosuppression. Nature Reviews Nephrology [Internet]. 2010 Sep;6(9):511–9. Available from: http://www.nature.com/articles/nrneph.2010.102

For peer review only - http://bmjopen.bmj.com/site/about/guidelines.xhtml

3. Devine PA, Courtney AE, Maxwell AP. Cardiovascular risk in renal transplant recipients. Journal of Nephrology [Internet]. 2019 Jun 7;32(3):389–99. Available from: http://link.springer.com/10.1007/s40620-018-0549-4

4. Weiner DE, Carpenter MA, Levey AS, Ivanova A, Cole EH, Hunsicker L, et al. Kidney function and risk of cardiovascular disease and mortality in kidney transplant recipients: The FAVORIT trial. American Journal of Transplantation. 2012 Sep;12(9):2437–45.

5. Karuthu S, Blumberg EA. Common Infections in Kidney Transplant Recipients. Clinical Journal of the American Society of Nephrology [Internet]. 2012 Dec;7(12):2058–70. Available from: https://cjasn.asnjournals.org/lookup/doi/10.2215/CJN.04410512

6. Kawai K, Uchiyama M, Hester J, Wood K, Issa F. Regulatory T cells for tolerance. Human Immunology [Internet]. 2018 May;79(5):294–303. Available from: https://linkinghub.elsevier.com/retrieve/pii/S0198885917305785

7. Romano M, Fanelli G, Albany CJ, Giganti G, Romano M, Lombardi G. Past , Present , and Future of Regulatory T Cell Therapy in Transplantation and Autoimmunity. 2019;10(January).

8. Tang Q, Lee K. Regulatory T-cell therapy for transplantation. Current Opinion in Organ Transplantation [Internet]. 2012 Aug;17(4):349–54. Available from: http://journals.lww.com/00075200-201208000-00005

9. Issa F, Hester J, Milward K, Wood KJ. Homing of Regulatory T Cells to Human Skin Is Important for the Prevention of Alloimmune-Mediated Pathology in an In Vivo Cellular Therapy Model. Kassiotis G, editor. PLoS ONE [Internet]. 2012 Dec 31;7(12):e53331. Available from: https://dx.plos.org/10.1371/journal.pone.0053331

10. Wu DC, Hester J, Nadig SN, Zhang W, Trzonkowski P, Gray D, et al. Ex Vivo Expanded Human Regulatory T Cells Can Prolong Survival of a Human Islet Allograft in a Humanized Mouse Model. Transplantation [Internet]. 2013 Oct 27;96(8):707–16. Available from: https://journals.lww.com/00007890-201310270-00006

11. Nadig SN, Więckiewicz J, Wu DC, Warnecke G, Zhang W, Luo S, et al. In vivo prevention of transplant arteriosclerosis by ex vivo–expanded human regulatory T cells. Nature Medicine [Internet]. 2010 Jul 16;16(7):809–13. Available from: http://www.nature.com/articles/nm.2154

12. Todo S, Yamashita K, Goto R, Zaitsu M, Nagatsu A, Oura T, et al. A pilot study of operational tolerance with a regulatory T-cell-based cell therapy in living donor liver transplantation. Hepatology [Internet]. 2016 Aug;64(2):632–43. Available from: https://onlinelibrary.wiley.com/doi/10.1002/hep.28459

13. Harden PN, Game DS, Sawitzki B, van der Net JB, Hester J, Bushell A, et al. Feasibility, long-term safety, and immune monitoring of regulatory T cell therapy in living donor kidney transplant recipients. American Journal of Transplantation. 2020;

14. Sawitzki B, Harden PN, Reinke P, Moreau A, Hutchinson JA, Game DS, et al. Regulatory cell therapy in kidney transplantation (The ONE Study): a harmonised design and analysis of seven non-randomised, single-arm, phase 1/2A trials. The Lancet [Internet]. 2020 May;395(10237):1627–39. Available from: https://linkinghub.elsevier.com/retrieve/pii/S0140673620301677

For peer review only - http://bmjopen.bmj.com/site/about/guidelines.xhtml

15. Roemhild A, Otto NM, Moll G, Abou-El-Enein M, Kaiser D, Bold G, et al. Regulatory T cells for minimising immune suppression in kidney transplantation: phase I/IIa clinical trial. BMJ [Internet]. 2020 Oct 21;m3734. Available from: https://www.bmj.com/lookup/doi/10.1136/bmj.m3734

16. Levitsky J, Leventhal JR, Miller J, Huang X, Chen L, Chandrasekaran D, et al. Favorable effects of alemtuzumab on allospecific regulatory T-cell generation. Human Immunology [Internet]. 2012 Feb;73(2):141–9. Available from: https://linkinghub.elsevier.com/retrieve/pii/S0198885911005817

17. Fraser H, Safinia N, Grageda N, Thirkell S, Lowe K, Fry LJ, et al. A Rapamycin-Based GMP-Compatible Process for the Isolation and Expansion of Regulatory T Cells for Clinical Trials. Molecular Therapy - Methods and Clinical Development [Internet]. 2018 Mar 16 [cited 2019 May 14];8:198–209. Available from: http://www.ncbi.nlm.nih.gov/pubmed/29552576

18. Ekberg H, Tedesco-Silva H, Demirbas A, Vítko Š, Nashan B, Gürkan A, et al. Reduced Exposure to Calcineurin Inhibitors in Renal Transplantation. New England Journal of Medicine [Internet]. 2007 Dec 20;357(25):2562–75. Available from: http://www.nejm.org/doi/abs/10.1056/NEJMoa067411

19. Alemtuzumab-based induction treatment versus basiliximab-based induction treatment in kidney transplantation (the 3C Study): a randomised trial. The Lancet [Internet]. 2014 Nov;384(9955):1684–90. Available from: https://linkinghub.elsevier.com/retrieve/pii/S0140673614610953

20. Haynes R, Blackwell L, Staplin N, Herrington WG, Emberson J, Judge PK, et al. Campath, calcineurin inhibitor reduction, and chronic allograft nephropathy (the 3C Study) – results of a randomized controlled clinical trial. American Journal of Transplantation [Internet]. 2018 Jun 9;18(6):1424–34. Available from: https://onlinelibrary.wiley.com/doi/10.1111/ajt.14619

Figure Legend

Figure 1: Diagrammatic representation of the immunosuppressive regimen used in The TWO Study

Figure 2: Overview of maintenance immunosuppression dosing with minimisation in the TR001 (Cell therapy) arm

Figure 3: Key timepoints alongside clinical and immune monitoring plans

For peer review only - http://bmjopen.bmj.com/site/about/guidelines.xhtml

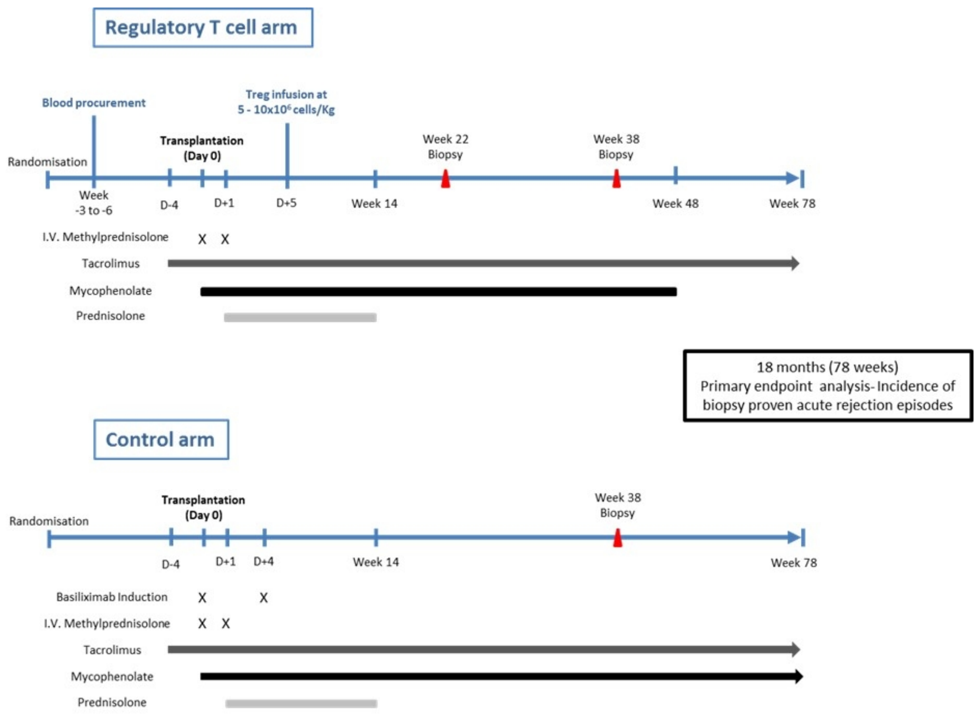

Diagrammatic representation of the immunosuppressive regimen used in The TWO Study

163x115mm (150 x 150 DPI)

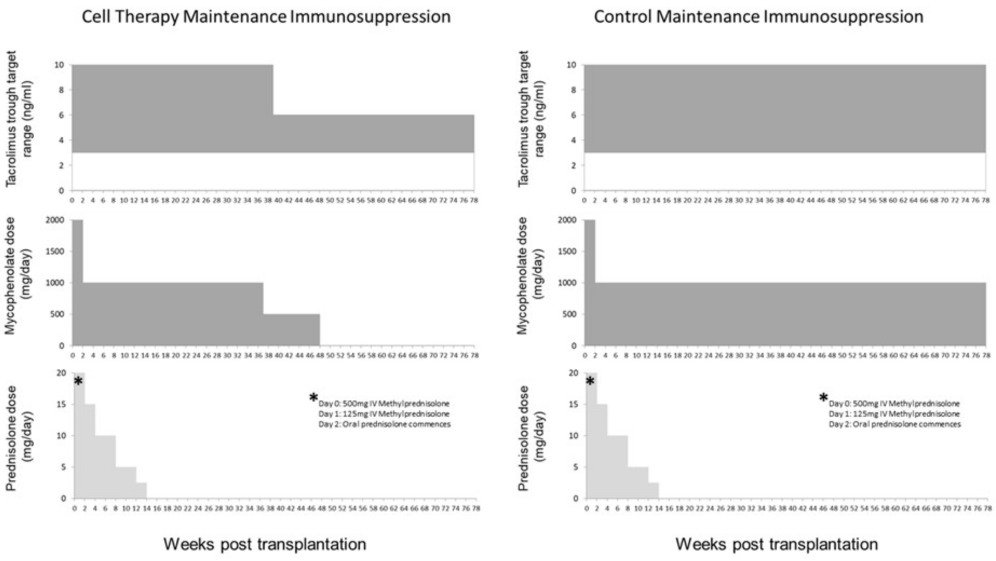

Overview of maintenance immunosuppression dosing with minimisation in the TR001 (Cell therapy) arm

156x88mm (150 x 150 DPI)

ASSESSMENTS	V0	V1	V2	Inpatient			V3	V4	V5	V6	V7	V8	V9	V10	V11	V12	V13	V14	V15	V16	V17	V18
	Pre-visit	min. - 3 weeks	W-2	D0	D4	D5	W1	W2	W4	W12	W22	W30	W38	W41	W43	W49	W51	W78	W104	W156	W208	W260
**STUDY GROUP**																						
Control	X		X	X	X		X	X	X	X		X	X		X		X	X	X	X	X	X
Intervention	X	X	X	X	X	X	X	X	X	X	X	X	X	X	X	X	X	X	X	X	X	X
**Rx ADMINISTRATION**																						
Treg/Cell isolation		X																				
TR001 (IMP) administration						X																
Basiliximab (Control arm)				X	X																	
**ASSESSMENTS**																						
EQ-5D-5L and SF-36 QOL questionnaire	X									X							X	X				
Clinical blood tests			X	X	X	X	X	X	X	X	X	X	X		X		X	X	X	X	X	X
Clinical urine test			X	X	X	X	X	X	X	X	X	X	X	X	X	X	X	X	X	X	X	X
Donor-Specific Antibodies			X								X		X					X	X	X	X	X
Renal biopsy											X		X									
**IMMUNE MONITORING**																						
Gene expression			X		X		X	X	X	X	X		X		X		X	X				
Leukocyte/serum profiling			X		X		X	X	X	X	X		X		X		X	X				
Functional assays			X						X						X			X				
HLA-DR					X		X	X	X													

Key timepoints alongside clinical and immune monitoring plans

271x136mm (300 x 300 DPI)

SPIRIT

STANDARD PROTOCOL ITEMS: RECOMMENDATIONS FOR INTERVENTIONAL TRIALS

SPIRIT 2013 Checklist: Recommended items to address in a clinical trial protocol and related documents*

Section/item	Item No	Description	Addressed on page number
**Administrative information**			
Title	1	Descriptive title identifying the study design, population, interventions, and, if applicable, trial acronym	1
Trial registration	2a	Trial identifier and registry name. If not yet registered, name of intended registry	1, 4
	2b	All items from the World Health Organization Trial Registration Data Set	Not covered in manuscript
Protocol version	3	Date and version identifier	11
Funding	4	Sources and types of financial, material, and other support	13
Roles and responsibilities	5a	Names, affiliations, and roles of protocol contributors	1, 11
	5b	Name and contact information for the trial sponsor	11
	5c	Role of study sponsor and funders, if any, in study design; collection, management, analysis, and interpretation of data; writing of the report; and the decision to submit the report for publication, including whether they will have ultimate authority over any of these activities	Not covered in manuscript
	5d	Composition, roles, and responsibilities of the coordinating centre, steering committee, endpoint adjudication committee, data management team, and other individuals or groups overseeing the trial, if applicable (see Item 21a for data monitoring committee)	Not covered in manuscript

For peer review only

For peer review only - http://bmjopen.bmj.com/site/about/guidelines.xhtml

**Introduction**			
Background and rationale	6a	Description of research question and justification for undertaking the trial, including summary of relevant studies (published and unpublished) examining benefits and harms for each intervention	3, 4
	6b	Explanation for choice of comparators	4
Objectives	7	Specific objectives or hypotheses	3
Trial design	8	Description of trial design including type of trial (eg, parallel group, crossover, factorial, single group), allocation ratio, and framework (eg, superiority, equivalence, noninferiority, exploratory)	4
**Methods: Participants, interventions, and outcomes**			
Study setting	9	Description of study settings (eg, community clinic, academic hospital) and list of countries where data will be collected. Reference to where list of study sites can be obtained	4
Eligibility criteria	10	Inclusion and exclusion criteria for participants. If applicable, eligibility criteria for study centres and individuals who will perform the interventions (eg, surgeons, psychotherapists)	5, 6
Interventions	11a	Interventions for each group with sufficient detail to allow replication, including how and when they will be administered	6, 7
	11b	Criteria for discontinuing or modifying allocated interventions for a given trial participant (eg, drug dose change in response to harms, participant request, or improving/worsening disease)	Not covered in manuscript
	11c	Strategies to improve adherence to intervention protocols, and any procedures for monitoring adherence (eg, drug tablet return, laboratory tests)	Not covered in manuscript
	11d	Relevant concomitant care and interventions that are permitted or prohibited during the trial	Not covered in manuscript
Outcomes	12	Primary, secondary, and other outcomes, including the specific measurement variable (eg, systolic blood pressure), analysis metric (eg, change from baseline, final value, time to event), method of aggregation (eg, median, proportion), and time point for each outcome. Explanation of the clinical relevance of chosen efficacy and harm outcomes is strongly recommended	7, 8, 9

For peer review only - http://bmjopen.bmj.com/site/about/guidelines.xhtml

Participant timeline	13	Time schedule of enrolment, interventions (including any run-ins and washouts), assessments, and visits for participants. A schematic diagram is highly recommended (see Figure)	Figures 1 & 3
Sample size	14	Estimated number of participants needed to achieve study objectives and how it was determined, including clinical and statistical assumptions supporting any sample size calculations	9, 10
Recruitment	15	Strategies for achieving adequate participant enrolment to reach target sample size	Not covered in manuscript
**Methods: Assignment of interventions (for controlled trials)**			
Allocation:			
Sequence generation	16a	Method of generating the allocation sequence (eg, computer-generated random numbers), and list of any factors for stratification. To reduce predictability of a random sequence, details of any planned restriction (eg, blocking) should be provided in a separate document that is unavailable to those who enrol participants or assign interventions	4
Allocation concealment mechanism	16b	Mechanism of implementing the allocation sequence (eg, central telephone; sequentially numbered, opaque, sealed envelopes), describing any steps to conceal the sequence until interventions are assigned	4
Implementation	16c	Who will generate the allocation sequence, who will enrol participants, and who will assign participants to interventions	4
Blinding (masking)	17a	Who will be blinded after assignment to interventions (eg, trial participants, care providers, outcome assessors, data analysts), and how	4
	17b	If blinded, circumstances under which unblinding is permissible, and procedure for revealing a participant's allocated intervention during the trial	N/A
**Methods: Data collection, management, and analysis**			
Data collection methods	18a	Plans for assessment and collection of outcome, baseline, and other trial data, including any related processes to promote data quality (eg, duplicate measurements, training of assessors) and a description of study instruments (eg, questionnaires, laboratory tests) along with their reliability and validity, if known. Reference to where data collection forms can be found, if not in the protocol	Figure 3

	18b	Plans to promote participant retention and complete follow-up, including list of any outcome data to be collected for participants who discontinue or deviate from intervention protocols	4, 5, 10
Data management	19	Plans for data entry, coding, security, and storage, including any related processes to promote data quality (eg, double data entry; range checks for data values). Reference to where details of data management procedures can be found, if not in the protocol	Not covered in manuscript
Statistical methods	20a	Statistical methods for analysing primary and secondary outcomes. Reference to where other details of the statistical analysis plan can be found, if not in the protocol	10
	20b	Methods for any additional analyses (eg, subgroup and adjusted analyses)	10
	20c	Definition of analysis population relating to protocol non-adherence (eg, as randomised analysis), and any statistical methods to handle missing data (eg, multiple imputation)	10
**Methods: Monitoring**			
Data monitoring	21a	Composition of data monitoring committee (DMC); summary of its role and reporting structure; statement of whether it is independent from the sponsor and competing interests; and reference to where further details about its charter can be found, if not in the protocol. Alternatively, an explanation of why a DMC is not needed	10
	21b	Description of any interim analyses and stopping guidelines, including who will have access to these interim results and make the final decision to terminate the trial	10
Harms	22	Plans for collecting, assessing, reporting, and managing solicited and spontaneously reported adverse events and other unintended effects of trial interventions or trial conduct	Not covered in manuscript
Auditing	23	Frequency and procedures for auditing trial conduct, if any, and whether the process will be independent from investigators and the sponsor	Not covered in manuscript
**Ethics and dissemination**			
Research ethics approval	24	Plans for seeking research ethics committee/institutional review board (REC/IRB) approval	11

Protocol amendments	25	Plans for communicating important protocol modifications (eg, changes to eligibility criteria, outcomes, analyses) to relevant parties (eg, investigators, REC/IRBs, trial participants, trial registries, journals, regulators)	Not covered in manuscript
Consent or assent	26a	Who will obtain informed consent or assent from potential trial participants or authorised surrogates, and how (see Item 32)	4
	26b	Additional consent provisions for collection and use of participant data and biological specimens in ancillary studies, if applicable	Not covered in manuscript
Confidentiality	27	How personal information about potential and enrolled participants will be collected, shared, and maintained in order to protect confidentiality before, during, and after the trial	Not covered in manuscript
Declaration of interests	28	Financial and other competing interests for principal investigators for the overall trial and each study site	11
Access to data	29	Statement of who will have access to the final trial dataset, and disclosure of contractual agreements that limit such access for investigators	11
Ancillary and post-trial care	30	Provisions, if any, for ancillary and post-trial care, and for compensation to those who suffer harm from trial participation	Not covered in manuscript
Dissemination policy	31a	Plans for investigators and sponsor to communicate trial results to participants, healthcare professionals, the public, and other relevant groups (eg, via publication, reporting in results databases, or other data sharing arrangements), including any publication restrictions	4, 11
	31b	Authorship eligibility guidelines and any intended use of professional writers	Not covered in manuscript
	31c	Plans, if any, for granting public access to the full protocol, participant-level dataset, and statistical code	11
**Appendices**			
Informed consent materials	32	Model consent form and other related documentation given to participants and authorised surrogates	Not covered in manuscript
Biological specimens	33	Plans for collection, laboratory evaluation, and storage of biological specimens for genetic or molecular analysis in the current trial and for future use in ancillary studies, if applicable	8, 9 & figure 3

For peer review only - http://bmjopen.bmj.com/site/about/guidelines.xhtml

*It is strongly recommended that this checklist be read in conjunction with the SPIRIT 2013 Explanation & Elaboration for important clarification on the items. Amendments to the protocol should be tracked and dated. The SPIRIT checklist is copyrighted by the SPIRIT Group under the Creative Commons "Attribution-NonCommercial-NoDerivs 3.0 Unported" license.

For peer review only

BMJ Open

Transplantation Without Overimmunosuppression (TWO) study protocol: A phase 2b randomised controlled single-centre trial of regulatory T cell therapy to facilitate immunosuppression reduction in living donor kidney transplant recipients

Journal:	*BMJ Open*
Manuscript ID	bmjopen-2022-061864.R1
Article Type:	Protocol
Date Submitted by the Author:	07-Mar-2022

Complete List of Authors:	Brook, Matthew; University of Oxford Nuffield Department of Surgical Sciences; Oxford Transplant Centre,<br>Hester, Joanna; University of Oxford Nuffield Department of Surgical Sciences<br>Petchey, William; Oxford Transplant Centre,<br>Rombach, Ines; University of Oxford Nuffield Department of Orthopaedics Rheumatology and Musculoskeletal Sciences<br>Dutton, Susan; University of Oxford Nuffield Department of Orthopaedics Rheumatology and Musculoskeletal Sciences<br>Bottomley, Matthew; University of Oxford Nuffield Department of Surgical Sciences; Oxford Transplant Centre,<br>Black, Joanna; University of Oxford Nuffield Department of Orthopaedics Rheumatology and Musculoskeletal Sciences<br>Abdul-Wahab, Seetha; Guy's Hospital, NIHR Biomedical Research Centre GMP unit; University of Oxford Nuffield Department of Surgical Sciences<br>Bushell, Andrew; University of Oxford Nuffield Department of Surgical Sciences<br>Lombardi, Giovanna; King's College London Faculty of Life Sciences and Medicine, Peter Gorer Department of Immunobiology; Guy's Hospital, NIHR Biomedical Research Centre GMP unit<br>Wood, Kathryn; University of Oxford Nuffield Department of Surgical Sciences<br>Friend, Peter; University of Oxford Nuffield Department of Surgical Sciences; Oxford Transplant Centre,<br>Harden, Paul; Oxford Transplant Centre,<br>Issa, Fadi; University of Oxford Nuffield Department of Surgical Sciences

**Primary Subject Heading**:	Renal medicine
Secondary Subject Heading:	Surgery
Keywords:	Transplant surgery < SURGERY, TRANSPLANT MEDICINE, Clinical trials < THERAPEUTICS, IMMUNOLOGY, Nephrology < INTERNAL MEDICINE

Transplantation Without Overimmunosuppression (TWO) study protocol: A phase 2b randomised controlled single-centre trial of regulatory T cell therapy to facilitate immunosuppression reduction in living donor kidney transplant recipients

Matthew Brook[1,2], Joanna Hester[1], William Petchey[2], Ines Rombach[3], Susan Dutton[3], Matthew Bottomley[1,2], Joanna Black[3], Seetha Abdul-Wahab[1,3], Andrew Bushell[1], Giovanna Lombardi[4,5], Kathryn Wood[1], Peter Friend[2], Paul Harden[2]*, Fadi Issa[1]*.

1. Transplantation Research & Immunology Group, Nuffield Department of Surgical Sciences, University of Oxford, John Radcliffe Hospital, Oxford, U.K.
2. Oxford Transplant Centre, Oxford University Hospitals NHS Foundation Trust, Churchill Hospital, Oxford, U.K.
3. Oxford Clinical Trials Research Unit, Nuffield Orthopaedic Centre, Windmill Road, Headington, Oxford, U.K.
4. NIHR Biomedical Research Centre GMP unit, Guy's Hospital, London, U.K.
5. Peter Gorer Department of Immunobiology, School of Immunology and Microbial Science, Kings College London, London, UK

*Equal contribution to work

Corresponding author:

Dr Matthew Brook, Oxford Kidney Unit, Churchill Hospital, Oxford, OX3 7LE

+447411521634

matthew.brook@ouh.nhs.uk

Keywords:

Regulatory T cell

Renal Transplantation

Immune Tolerance

Living Donors

Immunosuppression minimisation

Word Count: 3752

For peer review only - http://bmjopen.bmj.com/site/about/guidelines.xhtml

ABSTRACT

Introduction

Regulatory T cell (Treg) therapy has been demonstrated to facilitate long-term allograft survival in pre-clinical models of transplantation and may permit reduction of immunosuppression and its associated complications in the clinical setting. Phase 1 clinical trials have shown Treg therapy to be safe and feasible in clinical practice. Here we describe a protocol for the TWO Study, a phase 2b randomised control trial of Treg therapy in living donor kidney transplant recipients that will confirm safety and explore efficacy of this novel treatment strategy.

Methods and Analysis

60 patients will be randomised on a 1:1 basis to Treg therapy (TR001) or standard clinical care (Control). Patients in the TR001 arm will receive an infusion of autologous polyclonal ex-vivo expanded Tregs 5 days after transplantation instead of standard monoclonal antibody induction. Maintenance immunosuppression will be reduced over the course of the post-transplant period to low-dose tacrolimus monotherapy. Control participants will receive a standard basiliximab-based immunosuppression regimen with long-term tacrolimus and mycophenolate mofetil immunosuppression. The primary endpoint is biopsy proven acute rejection over 18 months; secondary endpoints include immunosuppression burden, chronic graft disfunction, and drug-related complications.

Ethics and Dissemination

Ethical approval has been provided by the NHS Health Research Authority South Central - Oxford A Research Ethics Committee (reference 18/SC/0054). The study also received authorisation from the UK MHRA and is being run in accordance with the principles of good clinical practice (GCP), in collaboration with the registered trials unit OCTRU. Results from the TWO Study will be published in peer-reviewed scientific/medical journals and presented at scientific/clinical symposia and congresses.

The TWO Study is registered on the ISRCTN registry (11038572).

For peer review only

For peer review only - http://bmjopen.bmj.com/site/about/guidelines.xhtml

STRENGTHS AND LIMITATIONS OF THIS STUDY

- Randomisation will provide a contemporary control group to compare outcomes following regulatory T cell therapy and immunosuppression minimisation
- Absence of an induction agent, Day 5 Treg infusion and protocol defined immunosuppression reduction to low dose tacrolimus monotherapy in the TR001 arm represents a significant reduction in pharmaceutical immunosuppression burden compared to standard care
- Comprehensive clinical and immune monitoring planned over an 18 month follow up will permit assessment of clinical safety and efficacy as well as exploration of markers of immune activation and tolerance
- This study may be limited through being an open label single centre trial
- As a phase 2b trial with small participant numbers in each arm this study is not powered to provide definitive proof of efficacy

INTRODUCTION

Kidney transplantation is the gold standard treatment for patients with end-stage kidney disease and is associated with excellent short-term outcomes with graft survival of greater than 95% for living donor transplant recipients at 1 year(1). However, there remains significant scope for improvement in long-term outcomes with progressive reduction in graft survival over time(1). Furthermore, outcomes are limited by the complications of immunosuppression such as life-threatening infection, increased cardiovascular disease risk, and malignancy(2–5). Novel treatments such as regulatory T cell (Treg) therapy may improve long-term patient and graft outcomes both by reducing immune mediated graft dysfunction and facilitating reduction of immunosuppression to minimise the associated side-effects(6–8)

Tregs are typically defined by expression of the cell surface markers being $CD4^{+}CD25^{+}$ and their constitutive expression of the master transcription factor FOXP3. Extensive pre-clinical models have demonstrated their potency at supressing rejection responses resulting in long-term allograft survival in the absence of pharmaceutical immunosuppression(9–11). The first steps in translation of Treg therapy into the clinical setting of organ transplantation were taken by Todo et al. who infused a Treg enriched cell product (less than 15% Treg) into liver transplant recipients(12). 7 of 10 patients were able to completely withdraw immunosuppression although 3 patients experienced rejection episodes. The low purity of Tregs in the infused cell product and incidence of spontaneous tolerance in liver transplant recipients makes interpretation of these results uncertain. In kidney transplantation, we have recently demonstrated successful infusion of autologous polyclonal Tregs into 12 patients recruited as part of the ONE Study consortium(13,14). This phase 1 trial used dose escalation from $3x10^{6}$ to $10x10^{6}$ Tregs/kg bodyweight infused at day 5 post-transplantation. Participants did not receive any monoclonal antibody induction therapy and were initially maintained on prednisolone, mycophenolate mofetil and tacrolimus. Immunosuppression was weaned over the course of the first year and 4 of 12 patients were ultimately successfully reduced to tacrolimus monotherapy. 4 year follow up demonstrated no episodes of rejection compared to a 21.1% rejection rate in a retrospective control cohort receiving standard care. Furthermore, there was a suggestion of reduced incidence of opportunistic CMV and BKV infections (13). Our ONE Study colleagues in Berlin infused 11 patients with autologous polyclonal Tregs in a dose escalation manner at day 7 post-transplant(15). 8 patients were weaned successfully to tacrolimus monotherapy. 3 of 11 patients experienced biopsy proven acute rejection, a rate similar to that seen in patients undergoing standard care(15). These studies have demonstrated

For peer review only - http://bmjopen.bmj.com/site/about/guidelines.xhtml

initial safety and feasibility of Treg therapy and provide justification for continuation into phase 2 trials(14).

The TWO study will build on our work performed as part of the ONE study consortium(14) to provide further evidence of safety and to explore efficacy of Treg therapy to facilitate immunosuppression reduction in living donor kidney transplant recipients.

The TWO study was originally conceived as a phase 2b randomised (1:1) control trial of Treg therapy versus standard care in 68 living donor kidney transplant recipients (ISRCTN: 11038572). Patients in both arms received standard alemtuzumab induction at the point of transplant to facilitate lymphodepletion with a view to optimising the environment into which Treg were later infused in favour of tolerance induction(16). Immunosuppression in the Treg arm was minimised to tacrolimus monotherapy in advance of cell infusion at 6 months post-transplant and compared to ongoing standard maintenance immunosuppression with tacrolimus and mycophenolate mofetil. Target tacrolimus levels were reduced in the cell therapy arm to 4-6 ng/mL from week 40 post-transplant. The primary outcome was incidence of biopsy proven acute rejection between 6 and 18 months post-transplant.

Nine patients were recruited to this protocol and seven transplanted prior to the emergence of the COVID-19 pandemic. Due to concerns related to an increased risk of severe COVID-19 in the setting of alemtuzumab lymphodepletion, the trial protocol was modified to one utilising basiliximab-based induction immunosuppression. Basiliximab is a widely used induction immunosuppressive agent that binds to and blocks CD25, the alpha chain of the IL-2 receptor, resulting in T cell suppression. Seven patients treated under the original protocol with alemtuzumab induction will be reported as a cohort demonstrating our experience of Treg administration in this context. The current protocol comparing Treg therapy to basiliximab based standard immunosuppression will recruit 60 participants, form the basis of the TWO study and is reported in detail here.

METHODS AND ANALYSIS

Patient and public involvement

Patients were involved in the design and conduct of the TWO Study. During development the proposed study was presented and discussed with a patient focus group to ensure that it addressed a relevant need to the transplant patient community. Methodology was discussed to ensure acceptability and address any concerns. A transplant recipient has joined the independent trial steering committee bringing an invaluable patient perspective to discussions. Once the trial has been published, participants will be informed of the outcomes directly and results will be distributed to relevant patient groups.

Study design

In this parallel group, phase IIb trial, 60 eligible living donor kidney transplant recipients will be recruited from that undergoing kidney transplantation at a single academic hospital (Oxford Transplant Centre, Churchill Hospital, Oxford, U.K.) and randomised on a 1:1 basis to receive a standard basiliximab based immunosuppressive regimen (Control Arm) or Treg infusion associated with immunosuppression reduction (TR001 Arm) (figure 1).

Participants will be approached and enrolled by the clinical PI or deputy following approval of listing for living donor kidney transplantation by the clinical multi-disciplinary team meeting. Randomisation is computer generated and performed by minimisation, with stratification for ethnicity and HLA-DR mismatch. Treatment allocation will be open-label as pre-transplant venesection of blood for Treg manufacture in those allocated to the TR001 arm is required and

For peer review only - http://bmjopen.bmj.com/site/about/guidelines.xhtml

it is not ethically appropriate to perform venesection in control patients prior to major surgery. Accordingly, outcome assessors and statisticians are not blinded.

With a relatively small patient sample size, the emergence of significant numbers of patient discontinuation in the trial may obscure the true outcome of this research. Discontinued participants may be replaced by the recruitment of additional patients. The decision to replace individual patients will ultimately be made by the Clinical PI on the basis that some unanticipated factor may influence the clinical outcome in terms of the primary endpoint.

Inclusion and Exclusion criteria

Inclusion and exclusion criteria for both kidney transplant recipient and donor are listed in Table 1. Specific to transplantation, exclusion criteria originally included a cRF of >40% and a history of previous transplant. These were subsequently amended to permit recipients with a cRF of <60% and to allow patients with a previous transplant to participate. ABO blood group incompatible transplants, the presence of a pre-transplant DSA, or a history of desensitisation continue to meet exclusion criteria to ensure those transplants with the highest immunological risk are not included in this phase IIb study.

Kidney Recipient Inclusion Criteria

A prospective kidney transplant recipient is eligible for enrolment into the study if all of the following inclusion criteria apply:

- Chronic renal insufficiency necessitating kidney transplantation and approved to receive a kidney allograft from a living donor
- Willing and able to give informed consent for participation in the trial
- Aged 18 years or above
- In the Investigator's opinion, is able and willing to comply with all trial requirements
- Able to commence the immunosuppressive regimen at the protocol-specified time point
- Female participants of child bearing potential and male participants whose partner is of child bearing potential must be willing to ensure that they or their partner use highly effective contraception during the first 18 months post-transplant (see section on Contraception)
- Willing to allow his or her General Practitioner and consultant, if appropriate, to be notified of participation in the trial.

Kidney Recipient Exclusion Criteria

The participant may not enter the trial if ANY of the following apply:

- ~~Patient has previously received any tissue or organ transplant~~*
- Known contraindication to the protocol-specified treatments or medications
- ABO blood group incompatible with donor
- Calculated reaction frequency (CRF) of >60%** within 6 months prior to transplant
- Previous treatment with any desensitisation procedure (with or without IVIg)
- Concomitant malignancy or history of malignancy within 5 years prior to planned study entry (excluding successfully treated non-metastatic basal or squamous cell carcinomas of the skin)
- Serologically positive for anti-HIV-1/2 Ab, HbsAg, anti-HBcAb, antiHCV Ab, anti-HTLV-1/2 Ab or syphilis (treponema palladium)

For peer review only - http://bmjopen.bmj.com/site/about/guidelines.xhtml

- Significant liver disease, defined as persistently elevated ALT levels >3 x upper limit of normal range (ULN)
- Any other significant disease or disorder which, in the opinion of the Investigator, may either put the participants at risk because of participation in the trial, or may influence the result of the trial, or the participant's ability to participate in the trial
- Participation in another clinical trial during the study or within 28 days prior to planned study entry
- Female participant who is pregnant, lactating or planning pregnancy during the course of the trial
- Psychological, familial, sociological, or geographical factors potentially hampering compliance with the study protocol and follow-up visit schedule
- Any form of substance abuse, psychiatric disorder, or other condition

*= Removed from exclusion criteria by substantial amendment
**=Changed from >40% by substantial amendment based on new information comparing cRF to historical PRA.

Kidney Donor Inclusion Criteria

A prospective donor is eligible if all of the following inclusion criteria apply:

- Eligible for live kidney donation
- Aged at least 18 years
- ABO blood group compatible with the organ recipient
- Willing to provide personal, medical and biological data for the trial analysis
- Willing and able to provide a blood sample for the immune monitoring assays
- Willing and able to give informed consent for participation in the trial

Kidney Donor Exclusion Criteria

If a prospective donor fulfils any of the following criteria, they are ineligible for the trial:

- Exposure to any investigational agents at the time of kidney donation, or within 28 days prior to kidney donation
- Any form of substance abuse, psychiatric disorder, or other condition that, in the opinion of the Investigator, may invalidate communication with the Investigator designated personnel
- Is a paired exchange donor
- Is an altruistic donor

Table 1: Inclusion and exclusion criteria for TWO study transplant recipients and donors

Control Arm

Participants in the control arm undergo planned living donor kidney transplantation with a standard basiliximab (anti-CD25) based immunosuppression protocol (figure 1). Briefly, patients will be pre-loaded with tacrolimus starting four days prior to transplantation and continued long-term aiming for trough levels of 3-10ng/ml. On the day of transplant patients

For peer review only - http://bmjopen.bmj.com/site/about/guidelines.xhtml

commence mycophenolate mofetil at an initial maintenance dose of 1000mg twice a day. 500mg of intravenous methylprednisolone and 20mg intravenous basiliximab are administered at induction. On day 1 post-transplant 125mg intravenous methylprednisolone is administered before ongoing oral prednisolone commences at 20mg once a day on day 2. A further 20mg of intravenous basiliximab is given on day 4 post-transplant. Maintenance immunosuppression on discharge thus consists of tacrolimus aiming for trough levels of 3-10ng/ml, mycophenolate mofetil 1000mg twice a day and prednisolone 20mg once a day. Mycophenolate mofetil is reduced to 500mg twice a day from 14 days post-transplant and continued long-term. Prednisolone is weaned to stop over 14 weeks resulting in dual maintenance therapy with mycophenolate mofetil and tacrolimus. Immunosuppression regimens and dose reductions in both arms are summarised in figure 2.

TR001 Arm

Patients recruited to the cell therapy arm attend for venesection of 370mls of whole blood a minimum of 3 weeks prior to planned transplantation to permit manufacture of the autologous Treg product (TR001). Following transport to the good manufacturing practice (GMP) unit at Guy's and St Thomas' Hospital, London, whole blood undergoes negative selection of $CD8^+$ cells and positive selection of $CD25^+$ cells resulting in enrichment of $CD4^+CD25^+FOXP3^+$ Treg (approx. 75% of total cells entering the expansion phase). Polyclonal expansion of cells is achieved through up to 3 rounds of stimulation with anti-CD3 and anti-CD28 bead stimulation in the presence of IL-2. Importantly, rapamycin is added to the culture conditions and has been shown to promote Treg stability and preferential expansion over contaminant populations. Full details of the expansion protocol have been described elsewhere(17). Following expansion, the final cell product is cryopreserved at a dose of 5-10x10^6 cells/kg body weight of the intended recipient in preparation for future infusion.

Living donor kidney transplantation occurs in line with standard clinical practice but with minimisation of immunosuppression from the outset in the TR001 arm. Initial maintenance immunosuppression with tacrolimus (Envarsus, Chiesi is the preferred long-acting sustained release formulation in both arms to avoid Treg toxicity that may occur at peak concentrations), mycophenolate mofetil and prednisolone is provided in an identical manner to those participants in the control arm. Importantly, where basiliximab is administered to control patients, those in the TR001 arm will receive no monoclonal induction agent at the time of transplantation. On day 5 post-transplant patients in the TR001 arm receive an infusion of 5-10x10^6 cells/kg of thawed autologous polyclonal Tregs administered in 100mls of 5% human albumin solution (HAS).

Planned reduction of maintenance immunosuppression in the TR001 arm will be dependent on stable biochemical transplant function. In the TR001 arm, protocol biopsies are performed for monitoring purposes at 22 weeks and 38 weeks post-transplant. Target trough tacrolimus levels are reduced from 3-10ng/ml to 3-6ng/ml at week 38 once biopsy results have been received. The maintenance dose of mycophenolate mofetil will be reduced to 250mg twice a day from week 37 post-transplant and stopped at 48 weeks post-transplant such that patients will subsequently continue on low-dose tacrolimus monotherapy as long-term maintenance (figure 2).

Primary Outcome

The primary outcome is incidence of biopsy-confirmed acute rejection (BCAR) in the 18-months post-transplantation. A diagnosis of BCAR can be made based on protocol driven or

For peer review only - http://bmjopen.bmj.com/site/about/guidelines.xhtml

clinically indicated 'for cause' biopsies. 'For cause' biopsies may be performed during follow-up at the discretion of the responsible clinician taking into account the full clinical picture and are typically triggered by an unexplained rise in serum creatinine as per standard NHS practice. Whenever rejection is suspected, a for-cause graft biopsy will always be offered and performed with the patient's permission. The results of for-cause biopsies will be available to the trial investigators and the outcome will be documented in the electronic database.

All biopsies performed will be reviewed and reported by the study pathologist using the internationally accepted Banff criteria. Whenever a biopsy is reported as suspicious for rejection or borderline changes, responsibility for a diagnosis of rejection lies with the treating physician.

Secondary Outcomes

A number of secondary outcomes are defined in order to assess the safety, feasibility and potential additive benefits of both cellular therapy and associated immunosuppression minimisation on the clinical course of recipients post-transplantation (Figure 3). These secondary outcomes will be continuously monitored throughout the 18 month follow-up period post transplantation unless otherwise stated and can be further defined as follows:

Indicators of influence of Treg administration on graft outcome

Impact on acute rejection: Time to first acute rejection episode; Severity of acute rejection episode based on response to treatment and histological scoring; Total immunosuppressive burden at the final trial visit; and Incidence of graft loss through rejection.

Success in reduction of immunosuppression: Proportion of patients on tacrolimus monotherapy at the end of the study

Prevention of chronic graft dysfunction: Assessment of renal impairment, chronic allograft dysfunction and/or interstitial fibrosis and tubular atrophy (IF/TA) assessed by clinical (impairment of eGFR) and histopathological (Banff staging) measures

Avoidance of drug-related complications by immunosuppressant reduction: Incidence of drug-related adverse events

Patient survival

Markers of over-suppression of the immune system

Incidence of serious and/or opportunistic infections (especially CMV, EBV and polyoma (BK) virus) and incidence of neoplasia.

Signs of chronic toxicity associated with infusion of cell products

Incidence of auto-immune disorders, anaemia, cytopaenias, or biochemical disturbances unrelated to the function of the transplanted kidney.

Patient quality of life

Patient quality of life will be measured in both arms of the study at pre-transplant baseline, 12 weeks, 51 weeks and 78 weeks post-transplant using SF-36 & EQ-5D-5L questionnaires.

Immune monitoring

A critical component of the TWO study is comprehensive assessment of the impact of Treg infusion on the recipient's immune repertoire and its capacity to respond to donor, third-party

For peer review only - http://bmjopen.bmj.com/site/about/guidelines.xhtml

For peer review only

and non-allogeneic stimuli. Importantly, these assays will include analysis of whole blood and transplant biopsy samples taken from patients in both arms of the study. Assays remain experimental and will not be used to influence clinical decision making in the TWO study. However, accumulating evidence suggests the potential for these tools in tailoring individualised immunosuppression regimens and we aim to identify those that might prove suitable for this purpose going forwards whilst providing important mechanistic information on a basic science level in the current study. Figure 3 provides an overview of immune monitoring assays being performed.

Absolute quantification of HLA-DR expression by peripheral blood monocytes is a useful and reproducible surrogate marker of innate immune responses. HLA-DR quantification will be performed by flow cytometry and interpreted using the following pre-determined ranges: Normal healthy controls >15,000 molecules per cell; immunodepression 15,000 – 8,000 molecules per cell; immunoparesis <8,000 molecules per cell.

Assays will be performed to investigate whether cell therapy shifts kidney transplant recipients towards a more tolerance-prone phenotype or away from a rejection-prone phenotype. Gene expression of a defined set of tolerance-associated genes in whole blood will be profiled by qPCR. Leucocyte subset profiling will be performed by flow cytometry to quantify immune cell subpopulations in patient peripheral blood. Donor-reactive T cell frequencies will be measured following co-culture of recipient T cells with stored donor derived antigen presenting cells using a CD154/137 assay. This assay will be performed before and after transplantation to enable an estimation of the pre-transplant frequency of donor-reactive T cells, and detection of post-transplant sensitisation against donor antigen. Treg frequencies in patient blood will be measured by epigenetic analysis of the Treg-specific demethylated region (TSDR) of the FOXP3 gene. Finally, cytokine and metabolic profiling will be performed assessing inflammatory and regulatory cytokines as well as low-molecular-weight metabolites to provide a picture of the dynamic changes that may take place in the immune response after cellular therapy and immunosuppression modification.

Histopathological samples will be taken at 5 months (protocol biopsy) in kidney transplant recipients randomised to the TR001 arm. This biopsy will confirm the ongoing safety of Treg therapy and ensure no evidence of subclinical rejection. A 9 month protocol biopsy will be performed in all participants including the control arm to allow a histological comparison of the impact of Treg therapy.

Sample size calculation

A standard anti-CD25 monoclonal antibody based immunosuppression protocol as used in this study would be expected to result in a biopsy proven acute rejection rate of approximately 12 to 20% over 18 months post-transplant. Ekberg et al. demonstrated that daclizumab induction with triple maintenance therapy of low-dose tacrolimus, myophenolate mofetil and corticosteroids resulted in acute rejection diagnoses in 12.3% of transplant recipients in the first year post-transplant, a significant improvement on comparable alternative regimens at the time(18). Recently, the 3C study reported a 16% acute rejection rate in the first 6 months of a basiliximab based immunosuppression regimen and a further 3% over the following 18 months up to 2 years post-transplant(19,20). There is little data on anticipated rejection rates in patients treated with Treg therapy. We reported in our phase 1 trial a rejection rate of 21.1% in a control cohort receiving basiliximab based immunosuppression compared with no rejection episodes in patients receiving Treg therapy over 60 weeks post-transplant(13). In contrast, Roemchild et al, demonstrated a rejection rate of 27% in patients treated with polyclonal Treg therapy and

For peer review only - http://bmjopen.bmj.com/site/about/guidelines.xhtml

22% in an identical control cohort(15). However, numbers were small in both studies and although both used autologous polyclonal Treg the manufacturing processes and quality control assessment of the final product differed.

The TWO Study is a phase 2b study aimed at proving the feasibility, ongoing safety and exploring the efficacy of Treg therapy to facilitate a reduction in standard immunosuppression. We aim to provide the data required for future phase 3 sample size calculations. Recruitment of 30 participants in each arm will allow us to estimate rejection rates in both arms with an anticipated 80% Wilson confidence interval width between 10-23%, depending on the observed rate.

Data analysis plan

This early phase study will report data using 20% statistical significance and 80% confidence intervals.

Two analysis sets will be defined:

- Intention to-treat population: all patients who signed informed consent and were transplanted will be analysed in the groups to which they were randomised
- Per-protocol population: all patients who signed informed consent, were transplanted and were treated according to protocol specifications.

Descriptive statistics will be used to describe the demographics between the treatment groups. Withdrawn patients will also be described fully. Comparative analysis will be undertaken to provide an indication as to whether a definitive phase 3 randomised trial would be appropriate.

For continuous variables, the difference in the means and the corresponding 80% confidence interval will be reported for each treatment group and overall. For continuous variables, t-tests unadjusted or multivariable linear models adjusted for important factors will be applied

For categorical variables, the number (and percentage) of patients in each category will be reported for each treatment group and overall. For categorical variables, chi-squared tests will be used for comparing treatment groups or multivariable logistic models adjusted for important factors.

The primary outcome is biopsy proven acute rejection episode and the time to first biopsy proven acute rejection will be analysed using survival analysis techniques. Kaplan-Meier survival curves will be presented graphically. Cox proportional Hazards models will be used both unadjusted and adjusted for important factors. The log-rank test will be used to identify significance. Acute rejection rates at 18 months will be reported for both groups and as a difference in proportions, alongside the hazard ratios and 80% confidence interval will be reported. Patients who have been withdrawn or lost-to follow-up will be censored at their last known rejection-free time. Analysis adjusting for competing risks of allograft failure or death will be considered.

No interim analyses are planned, but a data safety and monitoring committee (DSMC) will review descriptive summaries of accumulating data and make recommendations on trial termination or modification to the trial steering committee (TSC) based on these data. The independent members of the DSMC panel are chosen from those leading in the field of clinical transplantation and/or with experience of previous cell therapy trials in the ONE Study consortium. They will conduct a review of data at least annually at the discretion of the committee and will be informed of any SARs or SUSARs as they occur by e-mail notification. The DSMC charter is available from the TWO Study team.

For peer review only - http://bmjopen.bmj.com/site/about/guidelines.xhtml

ETHICS, GOVERNANCE AND DISSEMINATION

This manuscript is based on TWO Study protocol version 7.0 11Aug2020. The TWO Study has received ethical approval from NHS Health Research Authority South Central - Oxford A Research Ethics Committee (reference 18/SC/0054). In addition, the study has received authorisation from the UK MHRA.

All information, data and results obtained from the TWO Study are confidential. Agreement from the Sponsor and TSC will be required prior to the public disclosure of any study-related data.

The results from the TWO Study will be published in peer-reviewed scientific/medical journals and presented at scientific/clinical symposia and congresses.

The TWO Study is sponsored by the University of Oxford (ctrg@admin.ox.ac.uk).

Authors' contributions:

PJF is chief investigator

PH & FI are Co-Principal investigators

FI is the MRC grant holder

JB, MB, SD, PF, JH, PH, FI, GL, WP, IR & KW contributed to development of the initial protocol

JH has oversight of immune monitoring activities

All authors have contributed to amendments made to the initial study protocol

Acknowledgment:

This research was supported by the National Institute for Health Research (NIHR) Biomedical Research Centre based at Guy's and St Thomas' NHS Foundation Trust and King's College London. The views expressed are those of the authors and not necessarily those of the NHS, the NIHR or the Department of Health.

Funding statement:

This work was supported by Medical Research Council grant number MR/N027930/1.

Competing interests statement:

PH is an advisor to Sangamo Therapeutics

Envarsus long-acting sustained release tacrolimus is provided by Chiesi Ltd on a cost-neutral basis.

MJB has received an educational grant and speakers fees from Astellas UK (Ltd)

For peer review only - http://bmjopen.bmj.com/site/about/guidelines.xhtml

REFERENCES

1. Poggio ED, Augustine JJ, Arrigain S, Brennan DC, Schold JD. Long-term kidney transplant graft survival—Making progress when most needed. American Journal of Transplantation [Internet]. 2021 Aug 8;21(8):2824–32. Available from: https://onlinelibrary.wiley.com/doi/10.1111/ajt.16463

2. Rama I, Grinyó JM. Malignancy after renal transplantation: the role of immunosuppression. Nature Reviews Nephrology [Internet]. 2010 Sep;6(9):511–9. Available from: http://www.nature.com/articles/nrneph.2010.102

3. Devine PA, Courtney AE, Maxwell AP. Cardiovascular risk in renal transplant recipients. Journal of Nephrology [Internet]. 2019 Jun 7;32(3):389–99. Available from: http://link.springer.com/10.1007/s40620-018-0549-4

4. Weiner DE, Carpenter MA, Levey AS, Ivanova A, Cole EH, Hunsicker L, et al. Kidney function and risk of cardiovascular disease and mortality in kidney transplant recipients: The FAVORIT trial. American Journal of Transplantation. 2012 Sep;12(9):2437–45.

5. Karuthu S, Blumberg EA. Common Infections in Kidney Transplant Recipients. Clinical Journal of the American Society of Nephrology [Internet]. 2012 Dec;7(12):2058–70. Available from: https://cjasn.asnjournals.org/lookup/doi/10.2215/CJN.04410512

6. Kawai K, Uchiyama M, Hester J, Wood K, Issa F. Regulatory T cells for tolerance. Human Immunology [Internet]. 2018 May;79(5):294–303. Available from: https://linkinghub.elsevier.com/retrieve/pii/S0198885917305785

7. Romano M, Fanelli G, Albany CJ, Giganti G, Romano M, Lombardi G. Past , Present , and Future of Regulatory T Cell Therapy in Transplantation and Autoimmunity. 2019;10(January).

8. Tang Q, Lee K. Regulatory T-cell therapy for transplantation. Current Opinion in Organ Transplantation [Internet]. 2012 Aug;17(4):349–54. Available from: http://journals.lww.com/00075200-201208000-00005

9. Issa F, Hester J, Milward K, Wood KJ. Homing of Regulatory T Cells to Human Skin Is Important for the Prevention of Alloimmune-Mediated Pathology in an In Vivo Cellular Therapy Model. Kassiotis G, editor. PLoS ONE [Internet]. 2012 Dec 31;7(12):e53331. Available from: https://dx.plos.org/10.1371/journal.pone.0053331

10. Wu DC, Hester J, Nadig SN, Zhang W, Trzonkowski P, Gray D, et al. Ex Vivo Expanded Human Regulatory T Cells Can Prolong Survival of a Human Islet Allograft in a Humanized Mouse Model. Transplantation [Internet]. 2013 Oct 27;96(8):707–16. Available from: https://journals.lww.com/00007890-201310270-00006

11. Nadig SN, Więckiewicz J, Wu DC, Warnecke G, Zhang W, Luo S, et al. In vivo prevention of transplant arteriosclerosis by ex vivo–expanded human regulatory T cells. Nature Medicine [Internet]. 2010 Jul 16;16(7):809–13. Available from: http://www.nature.com/articles/nm.2154

For peer review only - http://bmjopen.bmj.com/site/about/guidelines.xhtml

12. Todo S, Yamashita K, Goto R, Zaitsu M, Nagatsu A, Oura T, et al. A pilot study of operational tolerance with a regulatory T-cell-based cell therapy in living donor liver transplantation. Hepatology [Internet]. 2016 Aug;64(2):632–43. Available from: https://onlinelibrary.wiley.com/doi/10.1002/hep.28459

13. Harden PN, Game DS, Sawitzki B, van der Net JB, Hester J, Bushell A, et al. Feasibility, long-term safety, and immune monitoring of regulatory T cell therapy in living donor kidney transplant recipients. American Journal of Transplantation. 2020;

14. Sawitzki B, Harden PN, Reinke P, Moreau A, Hutchinson JA, Game DS, et al. Regulatory cell therapy in kidney transplantation (The ONE Study): a harmonised design and analysis of seven non-randomised, single-arm, phase 1/2A trials. The Lancet [Internet]. 2020 May;395(10237):1627–39. Available from: https://linkinghub.elsevier.com/retrieve/pii/S0140673620301677

15. Roemhild A, Otto NM, Moll G, Abou-El-Enein M, Kaiser D, Bold G, et al. Regulatory T cells for minimising immune suppression in kidney transplantation: phase I/IIa clinical trial. BMJ [Internet]. 2020 Oct 21;m3734. Available from: https://www.bmj.com/lookup/doi/10.1136/bmj.m3734

16. Levitsky J, Leventhal JR, Miller J, Huang X, Chen L, Chandrasekaran D, et al. Favorable effects of alemtuzumab on allospecific regulatory T-cell generation. Human Immunology [Internet]. 2012 Feb;73(2):141–9. Available from: https://linkinghub.elsevier.com/retrieve/pii/S0198885911005817

17. Fraser H, Safinia N, Grageda N, Thirkell S, Lowe K, Fry LJ, et al. A Rapamycin-Based GMP-Compatible Process for the Isolation and Expansion of Regulatory T Cells for Clinical Trials. Molecular Therapy - Methods and Clinical Development [Internet]. 2018 Mar 16 [cited 2019 May 14];8:198–209. Available from: http://www.ncbi.nlm.nih.gov/pubmed/29552576

18. Ekberg H, Tedesco-Silva H, Demirbas A, Vítko Š, Nashan B, Gürkan A, et al. Reduced Exposure to Calcineurin Inhibitors in Renal Transplantation. New England Journal of Medicine [Internet]. 2007 Dec 20;357(25):2562–75. Available from: http://www.nejm.org/doi/abs/10.1056/NEJMoa067411

19. Alemtuzumab-based induction treatment versus basiliximab-based induction treatment in kidney transplantation (the 3C Study): a randomised trial. The Lancet [Internet]. 2014 Nov;384(9955):1684–90. Available from: https://linkinghub.elsevier.com/retrieve/pii/S0140673614610953

20. Haynes R, Blackwell L, Staplin N, Herrington WG, Emberson J, Judge PK, et al. Campath, calcineurin inhibitor reduction, and chronic allograft nephropathy (the 3C Study) – results of a randomized controlled clinical trial. American Journal of Transplantation [Internet]. 2018 Jun 9;18(6):1424–34. Available from: https://onlinelibrary.wiley.com/doi/10.1111/ajt.14619

Figure Legend

Figure 1: Diagrammatic representation of the immunosuppressive regimen used in The TWO Study

For peer review only - http://bmjopen.bmj.com/site/about/guidelines.xhtml

Figure 2: Overview of maintenance immunosuppression dosing with minimisation in the TR001 (Cell therapy) arm

Figure 3: Key timepoints alongside clinical and immune monitoring plans

For peer review only

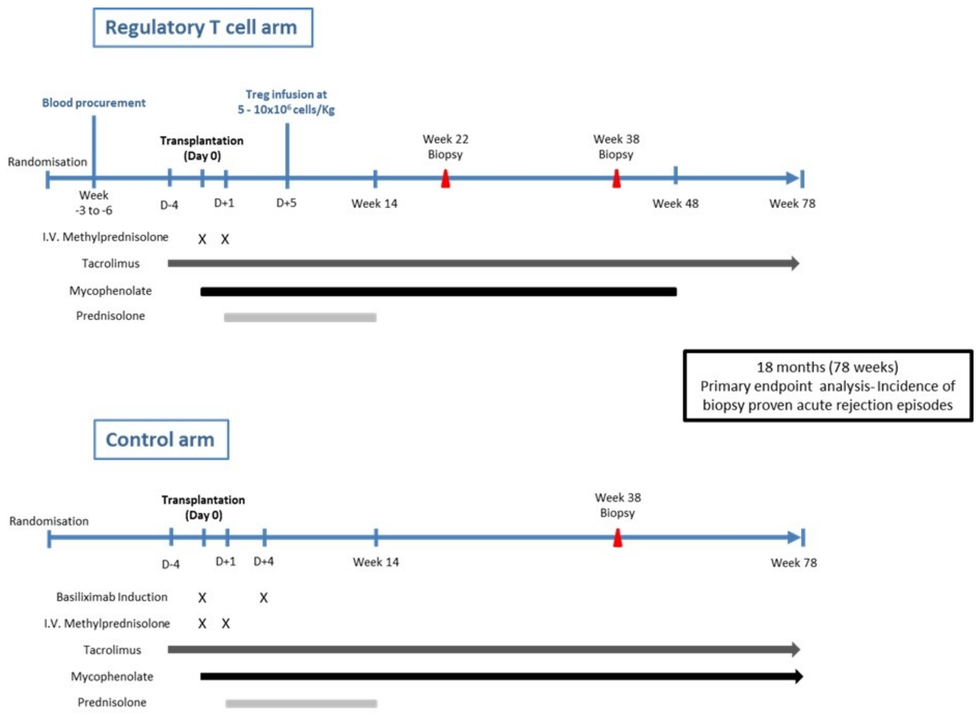

Diagrammatic representation of the immunosuppressive regimen used in The TWO Study

163x115mm (150 x 150 DPI)

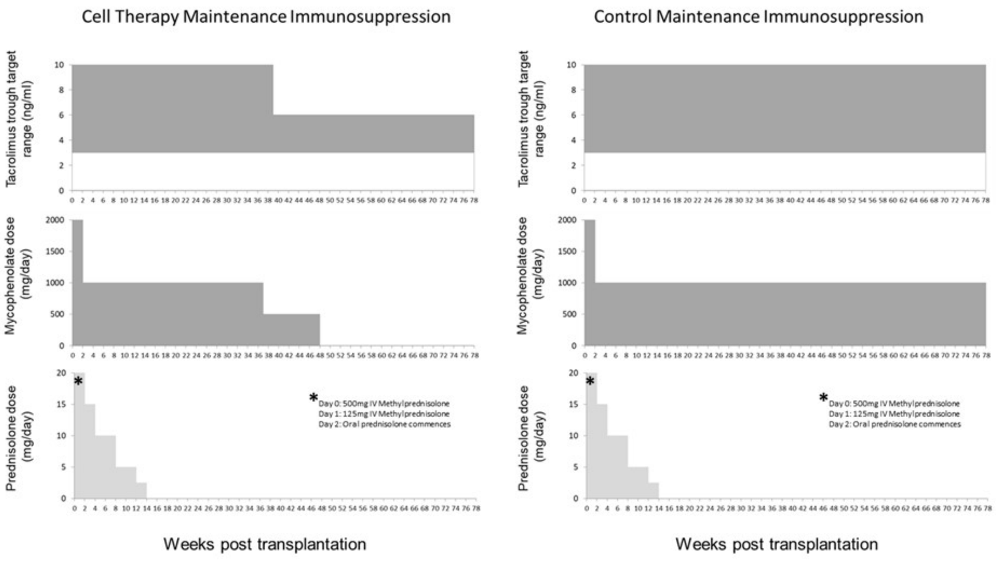

Overview of maintenance immunosuppression dosing with minimisation in the TR001 (Cell therapy) arm

156x88mm (150 x 150 DPI)

ASSESSMENTS	V0 Pre-visit	V1 min. - 3 weeks	V2 W-2	Inpatient D0	D4	D5	V3 W1	V4 W2	V5 W4	V6 W12	V7 W22	V8 W30	V9 W38	V10 W41	V11 W43	V12 W49	V13 W51	V14 W78	V15 W104	V16 W156	V17 W208	V18 W260
**STUDY GROUP**																						
Control	X		X	X	X		X	X	X	X		X	X		X		X	X	X	X	X	X
Intervention	X	X	X	X	X	X	X	X	X	X	X	X	X	X	X	X	X	X	X	X	X	X
**Rx ADMINISTRATION**																						
Treg/Cell isolation		X																				
TR001 (IMP) administration						X																
Basiliximab (Control arm)				X	X																	
**ASSESSMENTS**																						
EQ-5D-5L and SF-36 QOL questionnaire	X									X							X	X				
Clinical blood tests			X	X	X	X	X	X	X	X	X	X	X		X		X	X	X	X	X	X
Clinical urine test			X	X	X	X	X	X	X	X	X	X	X	X	X	X	X	X	X	X	X	X
Donor-Specific Antibodies			X								X		X					X	X	X	X	X
Renal biopsy											X		X									
**IMMUNE MONITORING**																						
Gene expression			X		X		X	X	X	X	X		X		X		X	X				
Leukocyte/serum profiling			X		X		X	X	X	X	X		X		X		X	X				
Functional assays			X						X						X			X				
HLA-DR					X		X	X	X													

Key timepoints alongside clinical and immune monitoring plans

271x136mm (600 x 600 DPI)

STANDARD PROTOCOL ITEMS: RECOMMENDATIONS FOR INTERVENTIONAL TRIALS

SPIRIT 2013 Checklist: Recommended items to address in a clinical trial protocol and related documents*

Section/item	Item No	Description	Addressed on page number
**Administrative information**			
Title	1	Descriptive title identifying the study design, population, interventions, and, if applicable, trial acronym	1
Trial registration	2a	Trial identifier and registry name. If not yet registered, name of intended registry	1, 4
	2b	All items from the World Health Organization Trial Registration Data Set	Not covered in manuscript
Protocol version	3	Date and version identifier	11
Funding	4	Sources and types of financial, material, and other support	13
Roles and responsibilities	5a	Names, affiliations, and roles of protocol contributors	1, 11
	5b	Name and contact information for the trial sponsor	11
	5c	Role of study sponsor and funders, if any, in study design; collection, management, analysis, and interpretation of data; writing of the report; and the decision to submit the report for publication, including whether they will have ultimate authority over any of these activities	Not covered in manuscript
	5d	Composition, roles, and responsibilities of the coordinating centre, steering committee, endpoint adjudication committee, data management team, and other individuals or groups overseeing the trial, if applicable (see Item 21a for data monitoring committee)	Not covered in manuscript

For peer review only

**Introduction**			
Background and rationale	6a	Description of research question and justification for undertaking the trial, including summary of relevant studies (published and unpublished) examining benefits and harms for each intervention	3, 4
	6b	Explanation for choice of comparators	4
Objectives	7	Specific objectives or hypotheses	3
Trial design	8	Description of trial design including type of trial (eg, parallel group, crossover, factorial, single group), allocation ratio, and framework (eg, superiority, equivalence, noninferiority, exploratory)	4
**Methods: Participants, interventions, and outcomes**			
Study setting	9	Description of study settings (eg, community clinic, academic hospital) and list of countries where data will be collected. Reference to where list of study sites can be obtained	4
Eligibility criteria	10	Inclusion and exclusion criteria for participants. If applicable, eligibility criteria for study centres and individuals who will perform the interventions (eg, surgeons, psychotherapists)	5, 6
Interventions	11a	Interventions for each group with sufficient detail to allow replication, including how and when they will be administered	6, 7
	11b	Criteria for discontinuing or modifying allocated interventions for a given trial participant (eg, drug dose change in response to harms, participant request, or improving/worsening disease)	Not covered in manuscript
	11c	Strategies to improve adherence to intervention protocols, and any procedures for monitoring adherence (eg, drug tablet return, laboratory tests)	Not covered in manuscript
	11d	Relevant concomitant care and interventions that are permitted or prohibited during the trial	Not covered in manuscript
Outcomes	12	Primary, secondary, and other outcomes, including the specific measurement variable (eg, systolic blood pressure), analysis metric (eg, change from baseline, final value, time to event), method of aggregation (eg, median, proportion), and time point for each outcome. Explanation of the clinical relevance of chosen efficacy and harm outcomes is strongly recommended	7, 8, 9

For peer review only - http://bmjopen.bmj.com/site/about/guidelines.xhtml

Participant timeline	13	Time schedule of enrolment, interventions (including any run-ins and washouts), assessments, and visits for participants. A schematic diagram is highly recommended (see Figure)	Figures 1 & 3
Sample size	14	Estimated number of participants needed to achieve study objectives and how it was determined, including clinical and statistical assumptions supporting any sample size calculations	9, 10
Recruitment	15	Strategies for achieving adequate participant enrolment to reach target sample size	Not covered in manuscript
**Methods: Assignment of interventions (for controlled trials)**			
Allocation:			
Sequence generation	16a	Method of generating the allocation sequence (eg, computer-generated random numbers), and list of any factors for stratification. To reduce predictability of a random sequence, details of any planned restriction (eg, blocking) should be provided in a separate document that is unavailable to those who enrol participants or assign interventions	4
Allocation concealment mechanism	16b	Mechanism of implementing the allocation sequence (eg, central telephone; sequentially numbered, opaque, sealed envelopes), describing any steps to conceal the sequence until interventions are assigned	4
Implementation	16c	Who will generate the allocation sequence, who will enrol participants, and who will assign participants to interventions	4
Blinding (masking)	17a	Who will be blinded after assignment to interventions (eg, trial participants, care providers, outcome assessors, data analysts), and how	4
	17b	If blinded, circumstances under which unblinding is permissible, and procedure for revealing a participant's allocated intervention during the trial	N/A
**Methods: Data collection, management, and analysis**			
Data collection methods	18a	Plans for assessment and collection of outcome, baseline, and other trial data, including any related processes to promote data quality (eg, duplicate measurements, training of assessors) and a description of study instruments (eg, questionnaires, laboratory tests) along with their reliability and validity, if known. Reference to where data collection forms can be found, if not in the protocol	Figure 3

For peer review only - http://bmjopen.bmj.com/site/about/guidelines.xhtml

	18b	Plans to promote participant retention and complete follow-up, including list of any outcome data to be collected for participants who discontinue or deviate from intervention protocols	4, 5, 10
Data management	19	Plans for data entry, coding, security, and storage, including any related processes to promote data quality (eg, double data entry; range checks for data values). Reference to where details of data management procedures can be found, if not in the protocol	Not covered in manuscript
Statistical methods	20a	Statistical methods for analysing primary and secondary outcomes. Reference to where other details of the statistical analysis plan can be found, if not in the protocol	10
	20b	Methods for any additional analyses (eg, subgroup and adjusted analyses)	10
	20c	Definition of analysis population relating to protocol non-adherence (eg, as randomised analysis), and any statistical methods to handle missing data (eg, multiple imputation)	10
**Methods: Monitoring**			
Data monitoring	21a	Composition of data monitoring committee (DMC); summary of its role and reporting structure; statement of whether it is independent from the sponsor and competing interests; and reference to where further details about its charter can be found, if not in the protocol. Alternatively, an explanation of why a DMC is not needed	10
	21b	Description of any interim analyses and stopping guidelines, including who will have access to these interim results and make the final decision to terminate the trial	10
Harms	22	Plans for collecting, assessing, reporting, and managing solicited and spontaneously reported adverse events and other unintended effects of trial interventions or trial conduct	Not covered in manuscript
Auditing	23	Frequency and procedures for auditing trial conduct, if any, and whether the process will be independent from investigators and the sponsor	Not covered in manuscript
**Ethics and dissemination**			
Research ethics approval	24	Plans for seeking research ethics committee/institutional review board (REC/IRB) approval	11

Protocol amendments	25	Plans for communicating important protocol modifications (eg, changes to eligibility criteria, outcomes, analyses) to relevant parties (eg, investigators, REC/IRBs, trial participants, trial registries, journals, regulators)	Not covered in manuscript
Consent or assent	26a	Who will obtain informed consent or assent from potential trial participants or authorised surrogates, and how (see Item 32)	4
	26b	Additional consent provisions for collection and use of participant data and biological specimens in ancillary studies, if applicable	Not covered in manuscript
Confidentiality	27	How personal information about potential and enrolled participants will be collected, shared, and maintained in order to protect confidentiality before, during, and after the trial	Not covered in manuscript
Declaration of interests	28	Financial and other competing interests for principal investigators for the overall trial and each study site	11
Access to data	29	Statement of who will have access to the final trial dataset, and disclosure of contractual agreements that limit such access for investigators	11
Ancillary and post-trial care	30	Provisions, if any, for ancillary and post-trial care, and for compensation to those who suffer harm from trial participation	Not covered in manuscript
Dissemination policy	31a	Plans for investigators and sponsor to communicate trial results to participants, healthcare professionals, the public, and other relevant groups (eg, via publication, reporting in results databases, or other data sharing arrangements), including any publication restrictions	4, 11
	31b	Authorship eligibility guidelines and any intended use of professional writers	Not covered in manuscript
	31c	Plans, if any, for granting public access to the full protocol, participant-level dataset, and statistical code	11
**Appendices**			
Informed consent materials	32	Model consent form and other related documentation given to participants and authorised surrogates	Not covered in manuscript
Biological specimens	33	Plans for collection, laboratory evaluation, and storage of biological specimens for genetic or molecular analysis in the current trial and for future use in ancillary studies, if applicable	8, 9 & figure 3

For peer review only - http://bmjopen.bmj.com/site/about/guidelines.xhtml

*It is strongly recommended that this checklist be read in conjunction with the SPIRIT 2013 Explanation & Elaboration for important clarification on the items. Amendments to the protocol should be tracked and dated. The SPIRIT checklist is copyrighted by the SPIRIT Group under the Creative Commons "Attribution-NonCommercial-NoDerivs 3.0 Unported" license.

For peer review only

For peer review only - http://bmjopen.bmj.com/site/about/guidelines.xhtml
