# Supplementary material for: Transplantation Without Overimmunosuppression (TWO) study protocol: a phase 2b randomised controlled single-centre trial of regulatory T cell therapy to facilitate immunosuppression reduction in living donor kidney transplant recipients
Source: BMJ Open. 2022 Apr 15;12(4):e061864. doi: 10.1136/bmjopen-2022-061864 (PMC9014059; doi:10.1136/bmjopen-2022-061864)
Supplement: Reviewer comments [file bmjopen-2022-061864.reviewer_comments.pdf]

## PEER REVIEW HISTORY

BMJ Open publishes all reviews undertaken for accepted manuscripts. Reviewers are asked to complete a checklist review form (**http://bmjopen.bmj.com/site/about/resources/checklist.pdf**) and are provided with free text boxes to elaborate on their assessment. These free text comments are reproduced below.

## ARTICLE DETAILS

| | |
|---|---|
| **TITLE (PROVISIONAL)** | Transplantation Without Overimmunosuppression (TWO) study protocol: A phase 2b randomised controlled single-centre trial of regulatory T cell therapy to facilitate immunosuppression reduction in living donor kidney transplant recipients |
| **AUTHORS** | Brook, Matthew; Hester, Joanna; Petchey, William; Rombach, Ines; Dutton, Susan; Bottomley, Matthew; Black, Joanna; Abdul-Wahab, Seetha; Bushell, Andrew; Lombardi, Giovanna; Wood, Kathryn; Friend, Peter; Harden, Paul; Issa, Fadi |

This article was not externally reviewed at BMJ Open. Protocols that have been independently assessed prior to submission to BMJ Open are usually fast-tracked to publication on the grounds that further substantial changes will not be possible. This independent assessment will usually be external, independent review for both a substantial grant award from a non-commercial or government funder and ethics approval.
